# Standardizing terms for clinical pharmacogenetic test results: consensus terms from the Clinical Pharmacogenetics Implementation Consortium (CPIC)

**DOI:** 10.1038/gim.2016.87

**Published:** 2016-07-21

**Authors:** Kelly E. Caudle, Henry M. Dunnenberger, Robert R. Freimuth, Josh F. Peterson, Jonathan D. Burlison, Michelle Whirl-Carrillo, Stuart A. Scott, Heidi L. Rehm, Marc S. Williams, Teri E. Klein, Mary V. Relling, James M. Hoffman

**Affiliations:** 1 Department of Pharmaceutical Sciences, St. Jude Children's Research Hospital, Memphis, Tennessee, USA; 2 Center for Molecular Medicine, NorthShore University HealthSystem, Evanston, Illinois, USA; 3 Department of Health Sciences Research, Mayo Clinic, Rochester, Minnesota, USA; 4 Department of Medicine, Vanderbilt University Medical Center, Nashville, Tennessee, USA; 5 Department of Biomedical Informatics, Vanderbilt University Medical Center, Nashville, Tennessee, USA; 6 Department of Genetics, Stanford University, Stanford, California, USA; 7 Department of Genetics and Genomic Sciences, Icahn School of Medicine at Mount Sinai, New York, New York, USA; 8 Brigham and Women's Hospital and Harvard Medical School, Boston, MA, USA; The Broad Institute of Harvard and MIT, Cambridge, Massachusetts, USA; 9 Genomic Medicine Institute, Geisinger Health System, Danville, Pennsylvania, USA

**Keywords:** CPIC, nomenclature, pharmacogenetics, pharmacogenomics, terminology

## Abstract

**Introduction:**

Reporting and sharing pharmacogenetic test results across clinical laboratories and electronic health records is a crucial step toward the implementation of clinical pharmacogenetics, but allele function and phenotype terms are not standardized. Our goal was to develop terms that can be broadly applied to characterize pharmacogenetic allele function and inferred phenotypes.

**Materials and methods:**

Terms currently used by genetic testing laboratories and in the literature were identified. The Clinical Pharmacogenetics Implementation Consortium (CPIC) used the Delphi method to obtain a consensus and agree on uniform terms among pharmacogenetic experts.

**Results:**

Experts with diverse involvement in at least one area of pharmacogenetics (clinicians, researchers, genetic testing laboratorians, pharmacogenetics implementers, and clinical informaticians; *n* = 58) participated. After completion of five surveys, a consensus (>70%) was reached with 90% of experts agreeing to the final sets of pharmacogenetic terms.

**Discussion:**

The proposed standardized pharmacogenetic terms will improve the understanding and interpretation of pharmacogenetic tests and reduce confusion by maintaining consistent nomenclature. These standard terms can also facilitate pharmacogenetic data sharing across diverse electronic health care record systems with clinical decision support.

*Genet Med* **19** 2, 215–223.

## Introduction

Many different terms are used to describe a variant allele's impact on enzyme function and the corresponding inferred phenotypic interpretation of a clinical pharmacogenetic test result. For example, a genetic testing laboratory report might interpret a TPMT *3A allele as leading to “low function,” “low activity,” “null allele,” “no activity,” or “undetectable activity.” Moreover, a laboratory might assign a phenotype designation to an individual carrying two nonfunctional *TPMT* alleles as being “TPMT homozygous deficient” while another laboratory might use the term “TPMT low activity.” These same laboratories could also use different terminology to describe a similar phenotype for a different gene (e.g., an individual carrying two nonfunctional *DPYD* alleles might be described as “DPYD defective”; see **Supplementary Tables S1 and S2** online). As a result, the use of inconsistent terms can be confusing to clinicians, laboratory staff, and patients. Although the actual phenotypes are the same in the *TPMT* and *DPYD* examples (i.e., no function), the terms describing these phenotypes have differed among laboratories and likely have led to confusion in the subsequent interpretation.

The lack of standard vocabularies describing pharmacogenetic results also interferes with the exchange of structured interpretations between laboratories, institutions using electronic health records (EHRs), and patients' personal health records. The impact on interoperability may significantly impede the portability of results throughout a patient's lifetime.^1,2,3^ Recently, a joint guideline was developed by the American College of Medical Genetics and Genomics (ACMG) and the Association for Molecular Pathology (AMP) that standardized the interpretation terms for describing the clinical significance of variants detected in Mendelian disease genes.^4^ ClinGen has utilized these terms to enable comparison of interpretations from clinical laboratories to identify and potentially resolve differences in variant interpretation,^5^ a critical step in improving the uniformity of patient care based on genetic information.

The Clinical Pharmacogenetics Implementation Consortium (CPIC) was formed in 2009 as a shared project between PharmGKB (https://www.pharmgkb.org) and the Pharmacogenomics Research Network (PGRN) (http://www.pgrn.org). CPIC provides clinical guidelines that enable the translation of pharmacogenetic laboratory test results into actionable prescribing decisions for specific drugs,^6^ which to date has produced 17 clinical guidelines (https://cpicpgx.org/genes-drugs). Currently, the terms used in CPIC guidelines to describe allele function and phenotype reflect community usage for each gene and are therefore not standard across CPIC guidelines (**Supplementary Table S3** online). Ideally, phenotype terms should be easily interpretable by clinicians with basic pharmacogenetic training and, when possible, should be consistent across genes encoding proteins with similar functions (e.g., the use of the term “poor metabolizer” could describe an individual carrying two nonfunctional alleles for any drug-metabolizing enzyme).

To maximize the utility of pharmacogenetic test results and to facilitate more uniform implementation of CPIC guidelines, it is essential to standardize these terms.^7^ To achieve this goal, particularly for purposes of clinical pharmacogenetic test reporting, CPIC initiated a project to identify terms that could be used consistently across pharmacogenes by developing a consensus among pharmacogenetics experts. The project participants used a modified Delphi method, which is a structured approach to establishing consensus through iterative surveys of an expert panel. When possible, the goal was to agree on uniform terms that could be applied across pharmacogenes to characterize (i) allele functional status and (ii) inferred phenotypes based on the combined impact of both alleles (i.e., diplotypes).

## Materials and Methods

The Delphi survey technique is an established approach to seeking expert consensus on a topic.^8,9,10^ The method uses a series of repeated structured questionnaires, or “rounds.” The rounds are usually anonymous and provide written, systematic refinement of expert opinion, and feedback of group opinion is provided after each round.^11^ Delphi survey technique guidelines proposed by Hasson et al. were consulted in the design of the project.^12^ The St. Jude Children's Research Hospital's institutional review board determined that this project does not meet the definition of research and was exempt from institutional review board purview.

**Figure 1:**
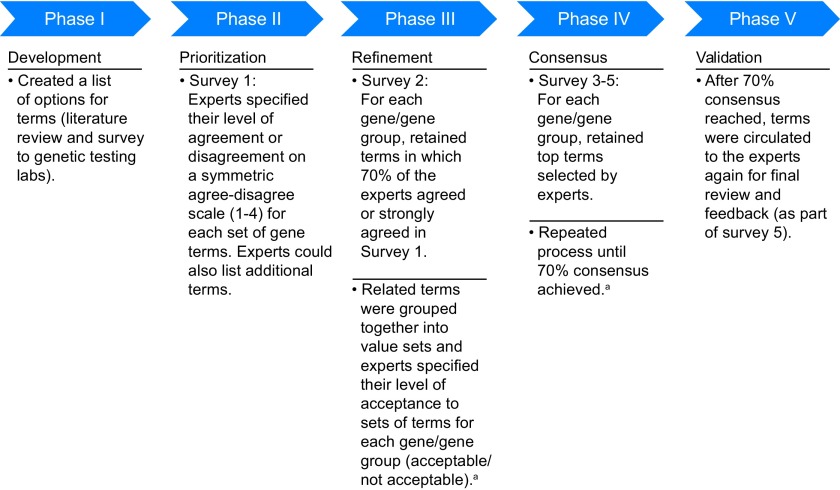
**Modified Delphi process.** ^a^Results from each prior survey were made available to the experts.

For the Delphi method used (**Figure 1**), CPIC solicited pharmacogenetic experts by e-mail invitation to members of CPIC, the PGRN, pharmacogenetic-related working groups for the Clinical Genome Resource (ClinGen; https://www.clinicalgenome.org), the Institute of Medicine DIGITizE Action Collaborative (http://iom.nationalacademies.org/Activities/Research/GenomicBasedResearch/Innovation-Collaboratives/EHR.aspx), the Centers for Disease Control and Prevention PGx nomenclature workgroup,^13^ the Global Alliance for Genomics and Health (GA4GH; http://ga4gh.org), ACMG (https://www.acmg.net), Electronic Medical Records and Genomics (eMERGE; https://emerge.mc.vanderbilt.edu), the CHAMP online resource for AMP members (http://champ.amp.org), and the College of American Pathologists (CAP).Experts not included in these groups were solicited by posting a description of the project on the PharmGKB website. All individuals who volunteered were included in survey 1.

Individuals were invited to participate in a series of surveys using an Internet-based survey tool (SurveyMonkey, Palo Alto, CA; http://www.surveymonkey.com) supplemented with live webinars that were used to explain the survey and solicit feedback. The webinars were designed to facilitate understanding of the survey to encourage completion; however, near the end of the process, an additional webinar was used to assist in developing a consensus. Each survey also included questions regarding the expert's workplace setting and degree of pharmacogenetic expertise (i.e., role in clinical pharmacogenetics and amount of time devoted to pharmacogenetics). 

Responses were included in the analysis if the respondents provided their name and contact information, which were necessary to enable follow-up with the respondents for the subsequent round (trainees were not excluded). Responses were tabulated as numeric counts and frequencies for each phase to determine whether consensus was reached. Analyses were also performed to determine whether there were differences in responses based on the expert's role in clinical pharmacogenetics. These analyses tested clinician versus nonclinician responses using chi-squared tests with an alpha of 0.05 to ensure that the final set of terms would be likely to be adopted by clinicians as well as laboratory-based researchers. All analyses were conducted in R version 3.0.1 (R Foundation for Statistical Computing, Vienna, Austria; http://www.R-project.org).

The goal of this project was to standardize terms used to characterize (i) allele functional status (i.e., allele descriptive terms) and (ii) inferred phenotypes based on the combined impact of both alleles (i.e., diplotypes). The terms used in the initial survey were identified by querying genetic testing laboratories and reviewing literature for currently used terms for CPIC Level A genes (https://cpicpgx.org/genes-drugs). This was informed by a literature review of references in the CPIC guidelines' evidence tables and the terms used in these papers to describe allele function and clinical phenotypes for genes with current CPIC guidelines (i.e., *CYP2D6*, *CYP2C19*, *CYP3A5*, *CYP2C9*, *TPMT*, *DPYD*, *HLA-B*, *UGT1A1*, *SLCO1B1*, and *VKORC1*) (**Supplementary Figures S1–S4** online). We also queried genetic testing laboratories listed at GeneTests (https://www.genetests.org/laboratories) and translational software companies and created a list of terms currently being used in laboratory reports.

For the first two survey rounds (surveys 1 and 2), terms that were found acceptable by at least 70% of the experts were retained for use in the next round. To improve semantic consistency, terms that were retained after survey 1 were assembled into value sets, which together described the range of possible descriptors of alleles or phenotypes. These value sets were evaluated in surveys 2 through 4, and the top value sets were retained until 70% consensus was reached. For surveys 1 and 2, genes that encode enzymes with similar metabolic function were combined when appropriate (e.g., *DPYD* and *TPMT* were combined, as were all the CYP enzymes excluding *CYP3A5*) and experts were given the opportunity to suggest alternative terms. In survey 1, experts were also asked how many categories of function/phenotype they felt were needed (e.g., three major categories for TPMT—high/normal, medium/some, no activity, versus five major categories for CYP enzymes). 

To promote consensus, a summary of comments from previous surveys was provided and experts were asked to read the comments prior to answering the questions (https://cpicpgx.org/resources/term-standardization). These comments were emphasized during the webinars to promote thoughtful discussion. Experts also had access to the full survey results. Of note, experts from surveys 1 and 2 commented in the survey and during webinar discussions that the standardized terms should be consistent across all pharmacogenes if possible. Based on this feedback and feedback from CPIC members, three categories of value sets were proposed and grouped together in survey 3: (i) drug-metabolizing enzymes (all CYP enzymes, UGT1A1, DPYD, and TPMT), (ii) drug transporters (e.g., SLCO1B1) and non–drug metabolizing enzymes (e.g., VKORC1), and (iii) high-risk genotypes (e.g., HLA-B). These groupings were used for the remainder of the surveys. Because consensus was not reached after survey 4, the experts were invited to participate in a conference call to discuss and recommend final terms, including consideration of the potential disruptive impact of adopting a new term for clinical laboratories versus any anticipated benefit of adopting a new term. These recommended terms were included in survey 5.

Although the Delphi method does not have a universal definition of consensus, 70% has been recommended and was considered a reasonable threshold given our diverse group of experts.^14,15^ Several new terms were added to survey 3 based on the feedback from rounds 1 and 2; these terms were built from existing terms and were included to improve semantic uniformity within a value set (**Supplementary Figures S1–S4** online). The final survey (survey 5) measured the level of acceptance of the final sets of terms. Results from each round were posted on PharmGKB (https://cpicpgx.org/resources/term-standardization) and were available to respondents throughout the process.

## Results

### Expert panel composition

A total of 222 individuals and approximately 2,000 subscribers to the CHAMP discussion board of AMP were invited to participate in the surveys; 58 completed survey 1, 54 completed survey 2, 47 completed survey 3, 46 completed survey 4, and 36 completed survey 5. The response group represented diverse involvement in at least one area of pharmacogenetics: 43% identified as clinicians, 67% as pharmacogenetics researchers, 19% as genetic testing laboratory staff, 43% as pharmacogenetics implementers, and 12% as clinical informaticians. In addition, 86% of the participants were from the United States, 10% from Europe, and 3% from other countries (i.e., Brazil and Egypt). Individuals were permitted to self-identify in more than one area; 48% of survey 1 respondents indicated that they spend >75% of their time devoted to pharmacogenetics, 57% of the experts were CPIC members, and 93% indicated they were involved in other pharmacogenetic-related groups (**Table 1**). See **Table 1** for additional demographics and numbers of experts for subsequent surveys.

*Phase 1: development.* Seven clinical testing laboratories submitted terms, and the results can be found in **Supplementary Tables S1 and S2** online. Terms identified in the literature review can be found in **Supplementary Table S3** online.

*Phase 2: prioritization.* Terms identified in phase 1 were used to create the first Delphi survey (survey 1) (see **Supplementary Tables S1–S3** online and **Supplementary Figures S1–S4** online for the complete list of terms). The prioritization phase was utilized to eliminate terms that experts found to be not appropriate. See https://cpicpgx.org/resources/term-standardization and **Supplementary Figures S1 and S2** online for results.

*Phases 3–5: refinement and consensus.* After survey 3, a consensus (77%) was reached for high-risk genotype genes but not for the other gene categories. Experts participating in survey 3 indicated that terms used to describe transporter function may not be suitable for all non-drug-metabolizing enzymes such as *VKORC1* or genes encoding drug receptors. Thus, *VKORC1* was excluded from future surveys (see “Discussion” for further explanation). Notably, assessing response rates between clinicians and nonclinicians did not reveal any significant differences (**Supplementary Figure S5** online).

At the conclusion of survey 4, one phenotype designation had not reached the targeted 70% consensus level. Although the phenotype designation of “intermediate metabolizer” was widely used in the literature to designate individuals between “normal metabolizer” and “poor metabolizer,” that term had not gained 70% consensus. After a conference call to discuss and recommend final terms to include in survey 5, and following completion of the final survey, 100% of experts agreed to terms for allele functional status for drug-metabolizing enzymes and transporters, 91.7% agreed to terms for drug-metabolizing enzyme phenotypes, and 91.7% agreed to terms for transporter phenotypes (**Supplementary Figure S6** online). The final terms and definitions are listed in **Table 2**.

## Discussion

We successfully engaged a diverse group of experts to establish standard terms through consensus for both pharmacogenetic allele function and inferred phenotypes. The final terms presented in **Table 2** will be used in all new and updated CPIC guidelines, and we recommend that these terms be considered standard terminology across all areas of clinical pharmacogenetics, including clinical genetic testing laboratory reporting. Moreover, these terms can be used for clinical decision support (CDS) to guide drug use and dosing (**Table 3**) using the suggested alerts in CPIC guidelines.^16,17,18,19^

In surveys 1 and 2 and during survey discussions, experts indicated that terms should be consistent across all genes if possible. Thus, terms describing phenotype were grouped together for subsequent surveys based on related enzyme functions. Final consensus terms included one set of terms to describe allele functional status and three sets of terms describing inferred phenotype depending on the type of pharmacogene: (i) drug-metabolizing enzymes (e.g., CYP2D6, DYPD, and TPMT), (ii) transporters (e.g., SLCO1B1), and (iii) high-risk genotypes (e.g., *HLA-B*) (**Table 2**). These terms are suitable for use in most CPIC level A and B genes (https://cpicpgx.org/genes-drugs).

Many experts felt that the historical and widely used term “extensive metabolizer” was too confusing for clinicians, often requiring clarification that it reflects “normal.” Therefore, the final consensus term “normal metabolizer” was selected, and “extensive metabolizer” will no longer be used in the CPIC guidelines. Furthermore, applying these standardized terms across all drug-metabolizing enzymes means that terms like “normal metabolizer” will also be used for genes such as *TPMT* and *DPYD* for which other designations were historically used (e.g., TPMT wild-type activity).

**Figure 2:**
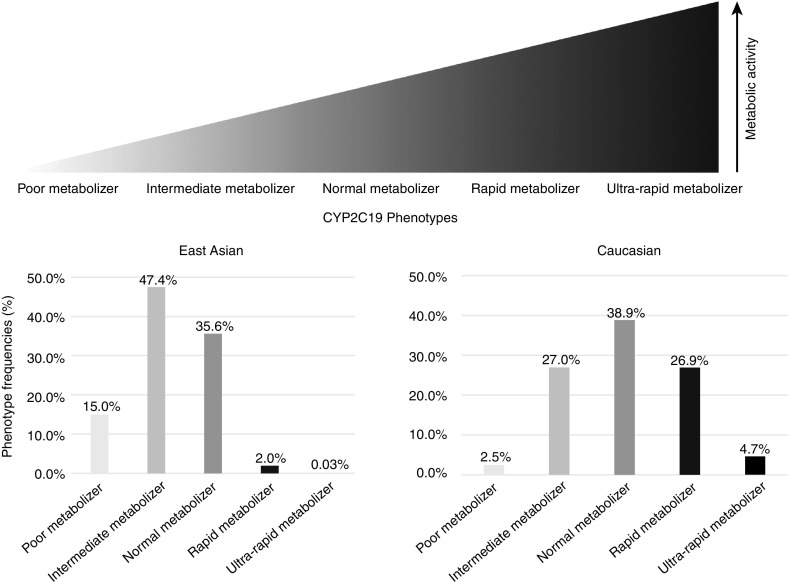
**Example of interpretive scale to visualize a drug metabolism gene's phenotype.** Phenotype frequencies were estimated using the equation describing Hardy-Weinberg equilibrium based on the allele frequencies published in the Clinical Pharmacogenetics Implementation Consortium guideline.^17^ For CYP2C19, phenotype frequencies differ substantially by ancestry. “Caucasian” includes those identified as European or North American in primary literature.

The speed with which we achieved consensus was based on the complexity of the gene and historical use of the term. Because of their simplicity and some level of standardization prior to this project, we quickly achieved consensus for the high-risk genotype genes (e.g., *HLA-B*). However, the phenotype terms describing drug-metabolizing enzymes were the most challenging to standardize owing to the different terms that have been used in research and clinical settings. Specifically, defining the term to distinguish the metabolizer status between “normal” and “poor” generated significant discussion. The panel eventually reached consensus on the commonly used term “intermediate metabolizer” after an additional review of the literature and after considering the difficulty of changing this specific term. Drug-metabolism terms often need to be interpreted considering the nature of the phenotypes relative to each other on a scale, going from very low function to very high function, which is more complex than expressing high-risk genotype genes as positive or negative for a specific variant allele. Visual depiction of such a scale (**Figure 2**) may be a helpful addition to interpretive reports.

Experts also had varying opinions about terms used to differentiate between alleles for which there is no literature describing function and alleles for which there are conflicting data to support the resulting function. In survey 2, the choices of terms were identical for “no literature describing function” and “conflicting data,” and experts chose different terms for each type of variant. Although the distinction may not be immediately apparent to clinical providers, we speculate that the experts differentiated these terms to be clear on the level and existence of evidence for a given variant. Distinguishing these concepts may provide value in certain contexts to distinguish lack of evidence from conflicting evidence, and this distinction may be emerging as a standard across genomic medicine (e.g., ClinVar).^20^

Additional standardization opportunities exist beyond the genes presented here. For example, *VKORC1* is the one CPIC level A gene (https://cpicpgx.org/genes-drugs) on which we did not reach a consensus. This gene is tested primarily in the context of predicting starting doses of the common anticoagulant warfarin, which is also dependent on *CYP2C9*. Therefore, many laboratories report a drug-centered phenotype such as “greatly increased sensitivity to warfarin” (see the CPIC guideline for warfarin^21^), which complicated standardization of *VKORC1* terms following the formats used for other genes. In addition, *VKORC1* genotype and inferred phenotypes for warfarin dosing are also reported by some laboratories and the CAP proficiency testing surveys according to the *CYP2C9* and *VKORC1* policy statement published by the ACMG in 2008,^22^ which further could have added to the difficulty in standardizing *VKORC1*.

This project and recent work^13^ have demonstrated that there is great diversity in how genetic test results are reported and interpreted,^23^ which can lead to confusion among clinicians, patients, and researchers in the exchange and use of clinical genetic data. Clear opportunities exist to develop new terminologies and improve existing standards to represent genetic results and interpretations.^24^ Although they do not represent comprehensive solutions, some progress has recently been made. An HL7 standard now exists that outlines how genetic test results could be reported.^25^ The Logical Observation Identifier Names and Codes (LOINC) terminology, a widely used standard for reporting laboratory test results and interpretations,^26,27^ is one terminology that could be used to report genetic interpretations, and it has recently been extended to support genetic data.^28^ Therefore, to enable precise communication beyond the CPIC guidelines, encourage use of these terms within EHRs, and facilitate the implementation of pharmacogenetic CDS, we obtained LOINC identifiers for pharmacogenetic interpretation codes and answer lists (**Supplementary Tables S4 and S5** online). Our work with LOINC has focused on standardizing pharmacogenetic test interpretation codes, and all the terms from the CPIC terminology-standardization project were registered as LOINC answer lists and were released on 21 December 2015 as part of LOINC 2.54.

The use of standardized vocabularies such as LOINC addresses a limitation identified in early implementations of pharmacogenetic CDS.^29^ Because pharmacogenetic expertise may remain concentrated in specialized healthcare centers but patients commonly move to and from a variety of healthcare providers, the consistent use of standard terms will improve the ability to share patient-specific pharmacogenetic knowledge across disparate clinical systems, including those systems with fewer resources for genomic medicine. In addition, the use of standard codes in CPIC guidelines to represent pharmacogenetic interpretation will facilitate further implementation of CDS rules, which are often triggered based on specific pharmacogenetic diagnoses with high-risk phenotypes.^29,30^

The Action Collaborative on Developing Guiding Principles for Integrating Genomic Information Into the Electronic Health Record Ecosystem (DIGITizE) (http://iom.nationalacademies.org/Activities/Research/GenomicBasedResearch/Innovation-Collaboratives/EHR.aspx), an ad hoc activity under the auspices of the Institute of Medicine Roundtable on Translating Genomic-Based Research for Health, engages key stakeholders from health information technology and management vendors, academic health centers, government agencies, and other organizations to work together to examine how genomic information can be uniformly represented and integrated into EHRs in a standards-based format. As an initial step, DIGITizE developed a CDS implementation guide for two pharmacogenetic use cases, HLA-B*57:01/abacavir and *TPMT*/azathioprine, based on the aforementioned HL7 standard and published CPIC guidelines. The implementation guide provides examples of HL7 messages for communicating the results of pharmacogenetic testing and CDS logic using the CPIC LOINC codes for HLA-B*57:01 and *TPMT*. As part of this effort, there was a careful decision to include only interpretations in the guide and not guidance for the genetic data itself. We anticipate that the availability of standard codes for pharmacogenetic interpretations will encourage the incremental development and dissemination of additional implementation resources.

In addition to facilitating LOINC implementation, another goal of CPIC is to have these standardized pharmacogenetic terms adopted broadly by clinical genetic testing laboratories and relevant professional societies and organizations. Importantly, after reviewing the CPIC term-standardization project and outcome, the AMP, which is an international society of more than 2,000 molecular and genomic laboratory medicine professionals, formally endorsed these pharmacogenetic terms on 26 October 2015 (http://www.amp.org/documents/AMPendorsementoftheCPICinitiative2015-10-26.pdf). The terms from this study also may have significant utility for collaborative genomic variation curation and interpretation efforts, including ClinGen and ClinVar.^31^ PharmGKB is currently working with ClinVar to deposit CPIC Level A gene/drug pairs using these standardized pharmacogenetic terms, and term adoption by other ClinVar submitters in the future would facilitate comparison across submissions. Additionally, these terms may be useful for proficiency testing programs that are designed to improve quality assurance and uniform pharmacogenetic interpretation among clinical genetic testing laboratories (e.g., College of American Pathologists (CAP-PGX)).

We chose to use a modified Delphi technique to build consensus among pharmacogenetic experts because it is an established and powerful tool to develop standards across different disciplines.^8,9,11^ Key risks to the validity of a Delphi study include overestimating the expertise of participants and attrition across the consensus rounds. Given that each participant had involvement in at least one area of pharmacogenetics and that 48% of survey 1 respondents indicated that they spend >75% of their time devoted to pharmacogenetics and 93% indicated they are involved with pharmacogenetic-related groups, we feel this is adequate support of the pharmacogenetic expertise among our survey participants. Participant attrition did occur across consensus rounds during our study; however, it was relatively low (**Table 1**) and determined to be nonsystematic. Although only 60% of the experts participated in survey 5, relative to other Delphi panels and the recommended minimum panel size, our final consensus panel was quite large, which reinforces the validity of our results.^32^ To reduce bias, especially the authority or reputation of specific individuals, Delphi panel participants are often kept anonymous throughout the process. Although survey creators and analysts were not blinded to participants, identifying information was not shared among survey participants. The only points of participant identification were between surveys when nonblinded e-mails were used to send invitations to conference calls and webinars during which interim results were discussed.

Because these terms were established by experts, an opportunity for further research is to formally assess the terms in end-user usability studies to understand their comprehension among clinicians and patients without formal training or experience in pharmacogenetics. The clinicians' specific practice site may influence their view of these terms. Although surveys of general populations of physicians have indicated limited knowledge and experience with pharmacogenetics^33,34^ and genome-guided prescribing through CDS,^35^ a more recent study conducted in a setting with a preemptive pharmacogenetics testing program revealed that their physicians were supportive of this type of program and that pharmacogenetic-guided therapy, particularly for cardiovascular medications, has clinical utility.^36^ Although our consensus terms were generated by experts, nearly 50% of our participants identified as clinicians, the use of terms by nonexpert clinicians and patients was considered throughout the process, and most of our experts practice in clinical settings with nonexperts.

We aimed to achieve consensus on acceptable terms for multiple pharmacogenes. On their own, these terms may not always be an adequate interpretation to guide clinicians, and additional interpretation information can be provided to set the observed phenotype in the context of other possible phenotypes. For example, with CYP enzymes, a normal metabolizer status would typically not trigger a dose that is different from that in the standard recommendation. However, in the case of tacrolimus, a CYP3A5 normal metabolizer (i.e., a CYP3A5 expresser) would require a higher recommended starting dose than the CYP3A5 poor metabolizer (a phenotype that is actually more common among those of European ancestry).^18^ In practice, it will be necessary to provide a patient's phenotypic designation in combination with other interpretive information designed for clinicians and patients, and various models of this approach already exist (**Table 3**).^37,38,39^

In conclusion, we anticipate that broad adoption of these proposed standardized pharmacogenetic terms will improve the understanding and interpretation of pharmacogenetic tests by clinicians and patients and reduce confusion by maintaining nomenclature consistency among pharmacogenes. Furthermore, these uniform references will reduce the complexity of the underlying coded vocabulary needed to transmit pharmacogenetic phenotypes between independent laboratories and sites of care and to trigger CDS.

## Figures and Tables

**Table 1:**
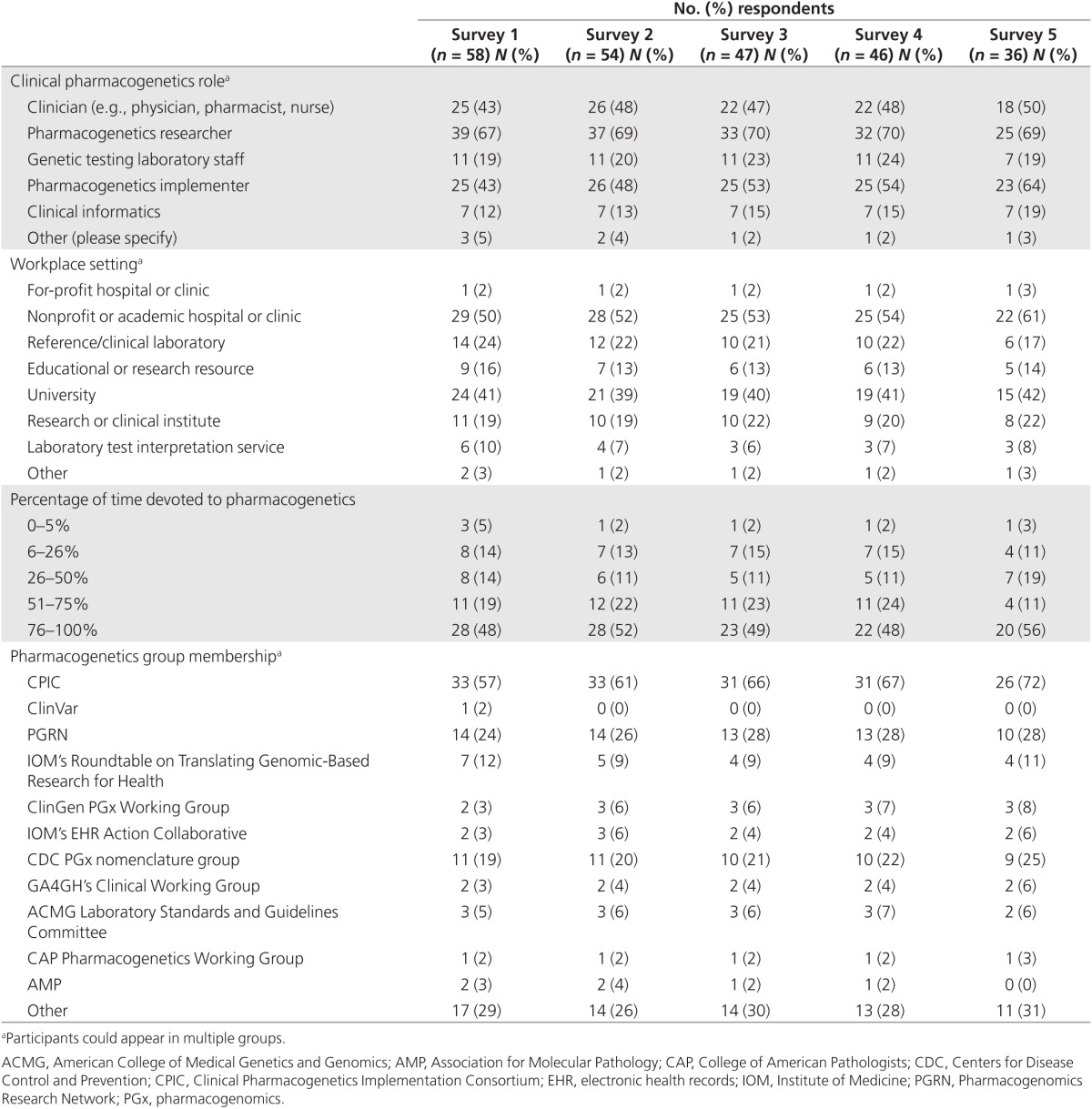
Demographics of experts

**Table 2:**
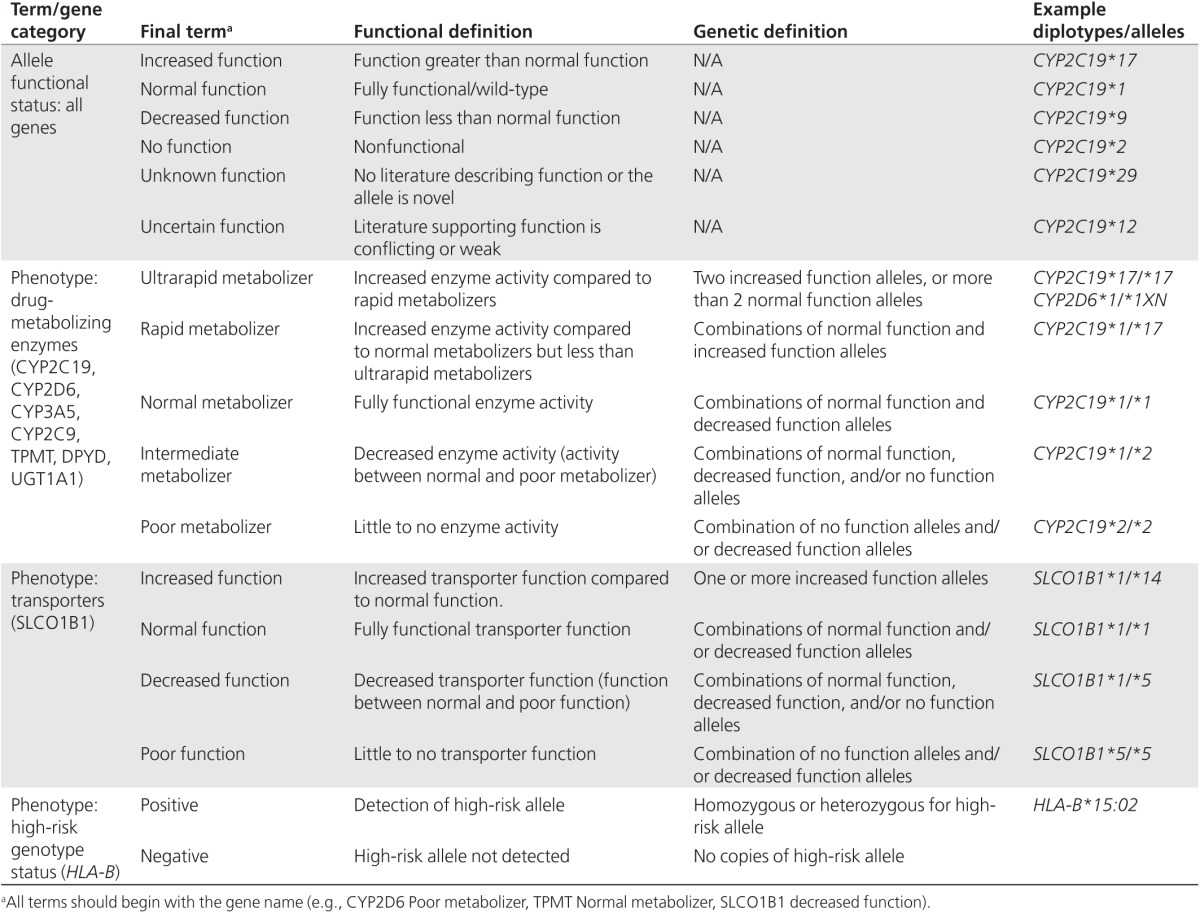
Final consensus terms for allele functional status and phenotype

**Table 3:**
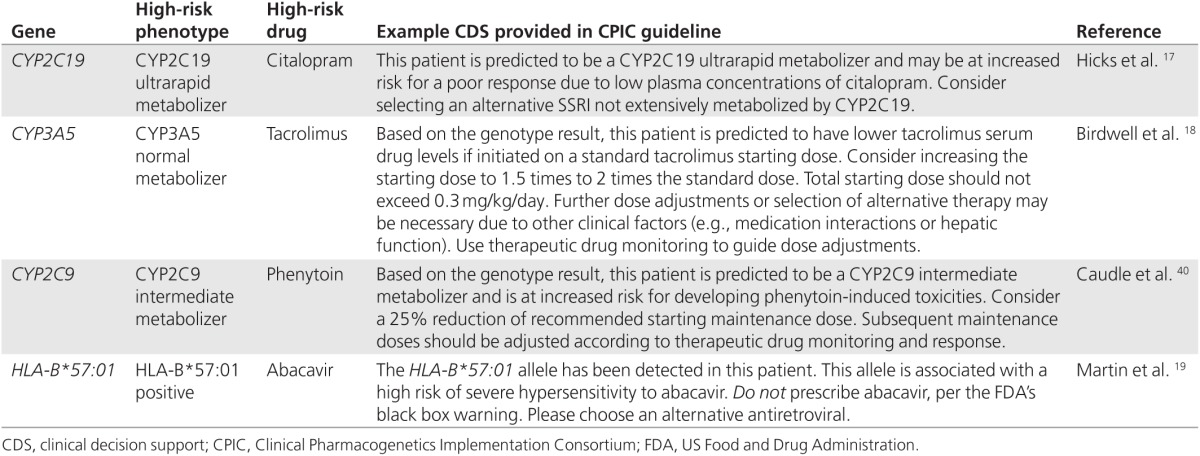
Examples of phenotype terms that trigger CDS
