# Supplementary material for: Standardizing terms for clinical pharmacogenetic test results: consensus terms from the Clinical Pharmacogenetics Implementation Consortium (CPIC)
Source: Genet Med. 2016 Jul 21;19(2):215–23. doi: 10.1038/gim.2016.87 (PMC5253119; doi:10.1038/gim.2016.87)
Supplement: Supplementary Materials [file gim201687x1.doc]

**Supplemental Material**

**Standardizing Terms For Clinical Pharmacogenetic Test Results: Consenus Terms from The Clinical PharmacogeneticS Implementation Consortium (CPIC)**

Authors: Kelly E. Caudle, Pharm.D., Ph.D.^1^, Henry M. Dunnenberger, Pharm.D.^2^, Robert R. Freimuth, Ph.D. ^3^, Josh F. Peterson, M.D. ^4^, Jonathan D. Burlison, Ph.D. ^1^, Michelle Whirl-Carrillo, Ph.D. ^5^, Stuart A. Scott, Ph.D. ^6^, Heidi L. Rehm, Ph.D. ^7^, Marc S. Williams, M.D. ^8^, Teri E. Klein, Ph.D. ^5^, Mary V. Relling, Pharm.D.^1^, James M. Hoffman, Pharm.D. M.S., ^1^

^1^Department of Pharmaceutical Sciences, St. Jude Children’s Research Hospital, Memphis, TN, USA

^2^Center for Molecular Medicine, NorthShore University HealthSystem, Evanston, IL, USA

^3^Department of Health Sciences Research, Mayo Clinic, Rochester, MN, USA

^4^Departments of Medicine and Biomedical Informatics, Vanderbilt University Medical Center, Nashville, TN, USA

^5^Department of Genetics, Stanford University, Stanford, CA, USA

^6^Department of Genetics and Genomic Sciences, Icahn School of Medicine at Mount Sinai, New York, NY, USA

^7^Brigham and Women’s Hospital and Harvard Medical School, Boston, MA, USA; The Broad Institute of Harvard and MIT, Cambridge, MA, USA

^8^Genomic Medicine Institute, Geisinger Health System, Danville, PA, USA

**Corresponding Author:**

Kelly E. Caudle, Pharm.D., Ph.D. BCPS

CPIC Coordinator

Pharmaceutical Sciences Department

St. Jude Children’s Research Hospital

262 Danny Thomas Place MS: 313

Memphis, TN 38105

Office: 901-595-3994

Cell: 901-289-7392

Kelly.caudle@stjude.org

Table of Contents

[Supplemental Table S1. Allele functional status terms used by six genetic testing laboratories at phase 1. 3](#__RefHeading___Toc455652979)

[Supplemental Table S2. Phenotype terms used by seven genetic testing laboratories at phase 1. 6](#__RefHeading___Toc455652980)

[Supplemental Table S3. Terms in the literature describing allele function and phenotype by gene at phase 1. 10](#__RefHeading___Toc455652981)

[Supplemental Figure S1. Terms used to describe allele functional status for drug metabolizing enzymes results by round 19](#__RefHeading___Toc455652982)

[Supplemental Figure S2. Terms used to describe allele functional status for non-drug metabolizing enzymes results by round 21](#__RefHeading___Toc455652983)

[Supplemental Figure S3. Terms used to describe phenotype for drug metabolizing enzymes results by survey round. 23](#__RefHeading___Toc455652984)

[Supplemental Figure S4. Terms used to describe phenotype for non-drug metabolizing enzymes results by survey round. 24](#__RefHeading___Toc455652985)

[Supplemental Table S4: LOINC pharmacogenetic interpretation codes 26](#__RefHeading___Toc455652986)

[Supplemental Table S5: LOINC answer lists for pharmacogenetic interpretation codes 27](#__RefHeading___Toc455652987)

[References 28](#__RefHeading___Toc455652988)

# Supplemental Table S1. Allele functional status terms used by six genetic testing laboratories at phase 1.

| **Gene** | **Variant Example** | **Lab B^a^** | **Lab C** | **Lab D** | **Lab E** | **Lab F** | **Lab G** |
| --- | --- | --- | --- | --- | --- | --- | --- |
| **Drug Metabolizing Enzymes** | | | | | | | |
| ***TPMT*** | **1* | Active | High/Normal activity |  |  |  |  |
|  | **8* | Partially active | No activity |  |  |  |  |
|  | **2* | Inactive | No activity |  |  |  |  |
| ***DPYD*** | **1* | Active | Normal activity |  |  |  |  |
|  | **3* | Partially active | Reduced activity |  |  |  |  |
|  | **2A* | Inactive | No activity |  |  |  |  |
| ***CYP2C19*** | **17* | Rapid | Increased function | Gain-of-function | Increased activity |  | Increased function |
|  | **1* | Active | Normal function | Normal function | Normal activity |  | Functional |
|  | **2* | Partially active | Loss-of-function | Non-functional | No activity |  | Decreased function |
|  | **3* | Inactive |  |  | No activity |  | Non-functional |
| ***CYP2D6*** | **1* (activity score 1) | Active | Normal function | Normal function | Normal activity |  | Functional |
|  | **10* (activity score 0.5) | Partially active | Reduced function | Reduced function | Reduced activity |  | Decreased function |
|  | **4* (activity score 0) | Inactive | No function | Non-functional | No activity |  | Non-functional |
|  | *1XN |  |  | Duplication |  |  | Increased function |
| ***CYP2C9*** | **1* | Active | Normal activity |  |  |  |  |
|  | **2* | Partially active | Decreased activity |  |  |  |  |
|  | **6* | Inactive | No activity |  |  |  |  |
| ***UGT1A1*** | **36* (5 TA repeats |  | Increased activity |  |  |  |  |
|  | **1* (6 TA repeats |  | Normal activity |  |  | Normal activity |  |
|  | **28* (7 TA repeats |  | Decreased activity |  |  | Reduced expression |  |
|  | **15* |  | No activity |  |  |  |  |
| ***CYP3A5*** | **1* | Active | Normal activity |  |  |  |  |
|  | **3* | Inactive | No activity |  |  |  |  |
| **Transporters** | | | | | | | |
| ***SLCO1B1*** | **14* | Rapid | High activity |  |  |  |  |
|  | **1* | Active | Normal activity |  |  |  |  |
|  | **9* | Partially active (*9) |  |  |  |  |  |
|  | **5* | Inactive (*5, etc.) | Low activity |  |  |  |  |
| **High-risk alleles** | | | | | | | |
| ***HLA-B*** | *HLA-B*15:02* present | Positive | Carrier |  |  |  |  |
|  | *HLA-B*15:02* not present | Negative | Non-carrier |  |  |  |  |
| ***VKORC1*** | **1* |  |  |  |  |  |  |
|  | *-1639G>A* |  | Decreased expression |  |  |  |  |

^a^Laboratory A does not report allele functional status.

# Supplemental Table S2. Phenotype terms used by seven genetic testing laboratories at phase 1.

| **Gene** | **Variant Example** | **Lab A** | **Lab B** | **Lab C** | **Lab D** | **Lab E** | **Lab F** | **Lab G** |
| --- | --- | --- | --- | --- | --- | --- | --- | --- |
| **Drug Metabolizing Enzymes** | | | | | | | | |
| ***TPMT*** | **1/*1* |  | Normal activity | Normal/High activity |  |  |  |  |
|  | **1/*2* |  | Intermediate activity | Intermediate activity |  |  |  |  |
|  | **3A/*3B* |  | Deficient activity | Deficient/Low activity |  |  |  |  |
| ***DPYD*** | **1* |  | Active | Normal activity |  |  |  |  |
|  | **3* |  | Partially Active | Reduced activity |  |  |  |  |
|  | **2A* |  | Inactive | No activity |  |  |  |  |
| ***CYP2C19*** | **17/*17* | Ultrarapid metabolizer | Ultra-rapid metabolizer | Rapid metabolizer | Ultra-rapid metabolizer | Rapid metabolizer |  | Ultra-rapid metabolizer |
|  | **1/*17* |  | Rapid metabolizer |  |  |  |  |  |
|  | **1/*1* | Extensive (Normal) metabolizer | Normal metabolizer | Normal metabolizer | Extensive metabolizer | Normal metabolizer |  | Normal metabolizer |
|  | **1/*3* | Intermediate metabolizer | Intermediate metabolizer | Intermediate metabolizer | Intermediate metabolizer | Intermediate metabolizer |  | Intermediate metabolizer |
|  | **3/*3* | Poor metabolizer | Poor metabolizer | Poor metabolizer | Poor metabolizer | Poor metabolizer |  | Poor metabolizer |
| ***CYP2D6*** | **1/*1xN* (activity score >2) | Ultrarapid metabolizer | Ultra Rapid metabolizer | Rapid metabolizer | Ultra-rapid metabolizer | Ultra-rapid metabolizer |  | Ultra-rapid metabolizer |
|  | **1/*1* (activity score 1-2) | Extensive (Normal) metabolizer | Normal | Normal metabolizer | Extensive metabolizer | Extensive metabolizer |  | Normal metabolizer |
|  | **4/*10* (activity score 0.5) | Intermediate metabolizer | Poor metabolizer | Intermediate metabolizer | Intermediate metabolizer | Intermediate metabolizer |  | Intermediate metabolizer |
|  | **4/*4* (activity score 0) | Poor metabolizer | Poor metabolizer | Poor metabolizer | Poor metabolizer | Poor metabolizer |  | Poor metabolizer |
| ***CYP2C9*** | **1/*1* | Extensive (Normal) metabolizer | Normal metabolizer | Normal metabolizer |  |  |  |  |
|  | **1/*2* | Intermediate metabolizer | Intermediate metabolizer | Intermediate metabolizer |  |  |  |  |
|  | **2/*2* | Poor metabolizer | Poor metabolizer | Poor metabolizer |  |  |  |  |
| ***UGT1A1*** | **1/*1* |  |  | Normal metabolizer |  |  |  |  |
|  | **1/*28* |  |  | Intermediate metabolizer |  |  | Not consistent with Gilbert phenotype but may result in elevated unconjugated bilirubin level |  |
|  | **28/*28* |  |  | Poor metabolizer |  |  | Consistent with Gilbert phenotype |  |
| ***CYP3A5*** | **1/*1* | Extensive metabolizer | Non-Expressers | Normal metabolizer |  |  |  |  |
|  | **1/*3* | Intermediate metabolizer | Intermediate expressers | Intermediate metabolizer |  |  |  |  |
|  | **3/*3* | Poor metabolizer | Expressers | Poor metabolizer |  |  |  |  |
| **Transporters** | | | | | | | | |
| ***SLCO1B1*** | *1/*1 |  | Normal transporter | Normal transporter function |  |  |  |  |
|  | *1/*5 |  | Intermediate transporter | Intermediate transporter function |  |  |  |  |
|  | *5/*5 |  | Poor transporter | Low transporter function |  |  |  |  |
| **High-risk alleles** | | | | | | | | |
| ***HLA-B*** | *HLA-B*15:02* present |  | Positive | carrier |  |  |  |  |
|  | *HLA-B*15:02* not present |  | Negative | non-carrier |  |  |  |  |
| ***VKORC1*** | AA | Greatly increased sensitivity | High sensitivity to warfarin | High sensitivity |  |  |  |  |
|  | GA | Increased sensitivity | Medium sensitivity to warfarin | Medium sensitivity |  |  |  |  |
|  | GG | Normal sensitivity | Low sensitivity to warfarin | Low sensitivity |  |  |  |  |

# Supplemental Table S3. Terms in the literature describing allele function and phenotype by gene at phase 1.

| **Gene** | | **CPIC functional status^a^** | | **Historical nomenclature (ref)** | | **CPIC phenotype^a^** | | **Phenotype definition** | | **Historical nomenclature (ref)** |
| --- | --- | --- | --- | --- | --- | --- | --- | --- | --- | --- |
| *TPMT* | | Functional / normal activity / wild-type | | Wild-type[^1-8^](#_ENREF_1)[_ENREF_8](#_ENREF_8)  High activity allele | | Homozygous wild-type or normal, high activity | | An individual carrying two or more functional (*1) alleles | | High activity  Homozygous wild-type[^12^](#_ENREF_12)  High methylator phenotype[^13^](#_ENREF_13) |
|  |  | Non-functional / variant / mutant / no activity | | Loss-of-function alleles[^4^](#_ENREF_4)  Mutant alleles  Low or undetectable levels of TPMT protein[^1^](#_ENREF_1)  Low activity allele  Variant alleles[^2^](#_ENREF_2)  Non-functional  Deficient activity  Decreased activity[^13^](#_ENREF_13) | | Heterozygote or intermediate activity | | An individual carrying one functional allele (*1) plus one non-functional allele | | Intermediate activity  Heterozygous deficient[^12^](#_ENREF_12)  Intermediate phenotype[^13^](#_ENREF_13)  Reduced TPMT activity |
|  |  |  | |  | | Homozygous variant, mutant, low, or deficient  activity | | An individual carrying two non-functional alleles | | Deficient activity  Very low or undetectable activity  Low activity  Homozygous TPMT deficient  Homozygous TPMT deficient[^12^](#_ENREF_12) |
| *CYP2C19* | | Functional / normal activity / wild-type | | Wild-type[^15-17^](#_ENREF_15)  Normal(http://www.cypalleles.ki.se/)  Normal function  Normal allele[^20^](#_ENREF_20) | | Ultra-rapid metabolizer (UM) | | An individual carrying two increased activity alleles or one functional allele plus one increased activity allele | | Ultra-rapid metabolizer[^21-27^](#_ENREF_21)  Non-wild type  Extensive metabolizer[^30-32^](#_ENREF_30)  Rapid metabolizer (*1/*17)[^25^](#_ENREF_25)  Ultra-metabolizer[^33^](#_ENREF_33) |
|  |  | Loss-of-function / no or decreased activity | | Deficient allele[^34^](#_ENREF_34)  Non-functional  Defective allele  Mutant allele[^15^](#_ENREF_15)  Decreased activity (http://www.cypalleles.ki.se/)  Decreased function  None (http://www.cypalleles.ki.se/)  Loss-of-function  Reduced-function allele  Absent function[^19^](#_ENREF_19) | | Extensive Metabolizer (EM) | | An individual carrying two functional alleles | | Homozygous extensive metabolizers  Extensive metabolizer  Wild-type  Rapid metabolizer[^63^](#_ENREF_63)  Normal metabolizer  Wild-type homozygote[^65^](#_ENREF_65)/homozygous[^66^](#_ENREF_66)  Homozygote normal metabolizer[^67^](#_ENREF_67) |
|  |  | Increased function / increased activity | | Increased activity  Ultra-rapid activity[^17^](#_ENREF_17)  Gain-of-function  Augmented function[^68^](#_ENREF_68) | | Intermediate Metabolizer (IM) | | An individual carrying one functional allele plus one loss-of-function allele or one loss-of-function allele plus one increased activity allele | | Heterozygous extensive metabolizers  Heterozygous metabolizer[^63^](#_ENREF_63)  Extensive metabolizer[^53^](#_ENREF_53)  Non-wild type  Intermediate metabolizer  Reduced metabolizer  Slow metabolizer[^64^](#_ENREF_64) |
|  |  |  | |  | | Poor Metabolizer (PM) | | An individual carrying two loss-of-function alleles | | Poor metabolizer  Reduced metabolizer  Slow metabolizer  Non-wild type |
| *CYP2D6* | Functional / normal activity/ wild-type | | Normal activity (http://www.cypalleles.ki.se/)  Wild-type activity  functional[^74^](#_ENREF_74) | | Ultra-rapid Metabolizer (UM) | | An individual carrying more than two copies of functional alleles | | Ultra-rapid metabolizer[^75-86^](#_ENREF_75)  Ultra-fast metabolizer | |
|  | Reduced-function / decreased activity | | Decreased activity (http://www.cypalleles.ki.se/)[^73^](#_ENREF_73)  Reduced activity | | Extensive Metabolizer (EM) | | An individual carrying two alleles encoding full or reduced function; or one full function allele together with either one non-functional or one reduced function allele | | Extensive metabolizer  Normal metabolizer[^87^](#_ENREF_87)  Homozygous extensive metabolizer[^85^](#_ENREF_85)  Heterozygous extensive metabolizer[^85^](#_ENREF_85) | |
|  | Non-functional, variant, or mutant / no activity | | None activity (http://www.cypalleles.ki.se/)  Defective allele[^73^](#_ENREF_73)  No activity[^72^](#_ENREF_72)  Null allele  Abolished activity[^73^](#_ENREF_73) | | Intermediate Metabolizer (IM) | | An individual carrying one reduced and one non-functional allele | | Intermediate metabolizer  Poor metabolizer[^78^](#_ENREF_78) | |
|  |  | |  | | Poor Metabolizer (PM) | | An individual carrying no functional alleles | | Poor metabolizer | |
| *DPYD* | Functional / normal activity/ wild-type | | Functional[^93^](#_ENREF_93)  Wild-type[^94^](#_ENREF_94) | | Homozygous wild-type or normal, high DPD activity | | An individual carrying two  or more functional (*1)  alleles | | Normal DPD activity[^95-97^](#_ENREF_95) | |
|  | Non-functional, variant, or mutant / no activity | | Null allele[^93^](#_ENREF_93)  Mutant allele  Non-functional  Low activity[^100^](#_ENREF_100)  Variant allele[^96^](#_ENREF_96)  No activity [^102^](#_ENREF_102)  Reduced activity[^102^](#_ENREF_102)  Decreased activity[^103^](#_ENREF_103)  Deficient[^103^](#_ENREF_103) | | Heterozygote or intermediate activity, partial DPD deficiency | | An individual carrying one  functional allele (*1) plus  one non-functional allele | | Reduced DPD activity  Decreased DPD activity  Low DPD activity  Partial DPD deficient | |
|  |  | |  | | Homozygous variant or mutant, DPD deficiency | | An individual carrying two  non-functional alleles | | Undetectable DPD activity[^100^](#_ENREF_100)  DPD deficient[^96^](#_ENREF_96)  Low activity[^99^](#_ENREF_99) | |
| *SLCO1B1* | Normal function | | Normal transport function  Wild-type | | Normal function or homozygous wild-type or normal | | An individual carrying two normal function alleles | | Homozygote wild-type[^108^](#_ENREF_108)  Common homozygote[^109^](#_ENREF_109)  Wild-type[^110-114^](#_ENREF_110)  Homozygous reference[^115^](#_ENREF_115)  Non-carrier[^116^](#_ENREF_116) | |
|  | Decreased function | | Decreased activity  Loss of activity [^119^](#_ENREF_119)  Reduced transport function  Low activity [^110^](#_ENREF_110)  Impaired transport activity  Reduced activity[^123^](#_ENREF_123)  Impaired function[^107^](#_ENREF_107)  Loss of function[^71^](#_ENREF_71) | | Intermediate function or heterozygous | | An individual carrying one normal function allele plus one decreased function allele | | Carriers[^107^](#_ENREF_107)  Heterozygote / heterozygous  Heterozygous carrier[^116^](#_ENREF_116)  Variant[^113^](#_ENREF_113) | |
|  | Increased function | | Gain in activity[^124^](#_ENREF_124)  Increased activity[^123^](#_ENREF_123) | |  | | An individual carrying two decreased function alleles | | Carriers[^107^](#_ENREF_107)  Homozygote variant[^108^](#_ENREF_108) / Homozygous variant[^112^](#_ENREF_112)  Rare homozygote[^109^](#_ENREF_109)  Mutant homozygous[^111^](#_ENREF_111)  Homozygous carrier[^116^](#_ENREF_116)  Variant[^113^](#_ENREF_113) | |
| *CYP2B6* |  | | Normal activity[^125^](#_ENREF_125)  Wild-type | |  | | An individual carrying two normal function alleles | | Extensive metabolizer  Homozygous Wild-type  Normal metabolizers  Wild-type | |
|  |  | | Accelerated metabolism[^145^](#_ENREF_145)  Enhanced activity[^126^](#_ENREF_126)  Enhanced metabolism[^146^](#_ENREF_146)  Gain of function  Higher metabolism[^149^](#_ENREF_149)  Increased expression  Increased activity  Increase specific activity  Ultra-rapid metabolizer variant[^133^](#_ENREF_133) | |  | | Carrier of at least one increase function allele | | Fast metabolizer[^135^](#_ENREF_135)  Heterozygous mutant  Heterozygous variant  Homozygous variant  Rapid metabolizer[^154^](#_ENREF_154)  Rare allele homozygous[^135^](#_ENREF_135)  Ultra-rapid metabolizer | |
|  |  | | Decreased activity  Decreased expression  Defective  Diminished activity[^164-166^](#_ENREF_164)  Diminished function[^146^](#_ENREF_146)  Loss of activity[^150^](#_ENREF_150)  Loss of function  Low activity  Nonfunctional  Undetectable activity[^145^](#_ENREF_145)  Reduced activity  Reduced expression[^179^](#_ENREF_179)  Reduced function Reduced metabolic capacity[^181^](#_ENREF_181) | |  | | Carrier of at least one decrease function allele | | Extensive metabolizer (only 1 reduced allele)  Heterozygote carrier[^53^](#_ENREF_53)  Heterozygous mutant  Heterozygous variant[^136^](#_ENREF_136)  Intermediate metabolizer  Low expresser[^150^](#_ENREF_150)  Poor metabolizer[^53^](#_ENREF_53)  Slow metabolizer | |
|  |  | |  | |  | | Carrier of two decrease function allele | | Deficiency[^157^](#_ENREF_157)  Homozygous mutant  Homozygous variant[^136^](#_ENREF_136)  Impaired metabolizer  Poor metabolizer  Rare allele homozygous[^135^](#_ENREF_135)  Slow metabolizer | |
| *CYP2C9* | Normal Activity | | Wild type[^190-193^](#_ENREF_190) | | Extensive metabolizer | | An individual carrying two  normal activity alleles | | Extensive metabolizer  Homozygous wild-type  Wild-type[^200-204^](#_ENREF_200)  Homozygous extensive metabolizer | |
|  | Decreased Activity | | Decreased activity  Decreased capacity[^207^](#_ENREF_207)  Impaired function[^208^](#_ENREF_208) Reduced activity  Reduced capacity[^209^](#_ENREF_209)  Reduced function[^193^](#_ENREF_193) | | Intermediate metabolizer | | An individual carrying one  normal activity allele plus  one decreased function allele | | Intermediate metabolizer  Extensive metabolizer[^53^](#_ENREF_53)  Slow metabolizer[^195^](#_ENREF_195)  Heterozygous poor metabolizer[^202^](#_ENREF_202)  Heterozygous carrier  Heterozygous extensive metabolizer | |
|  | Possible Decreased Activity | |  | | Poor metabolizer | | An individual carrying two  decreased function alleles | | Poor metabolizer  Slow metabolizer  Homozygous poor metabolizer[^202^](#_ENREF_202)  Homozygous carrier | |
|  | No Activity  Null | | Loss of function[^210^](#_ENREF_210) | |  | |  | |  | |
| *VKORC1* |  | | Warfarin resistance[^212^](#_ENREF_212) | |  | | An individual carrying two wild type allele/haplotypes | | Non-carriers[^212^](#_ENREF_212) | |
|  |  | | Wild type | |  | | An individual carrying one variant allele/haplotypes | | Heterozygotes Carriers | |
|  |  | | Warfarin sensitive[^190^](#_ENREF_190)  Decreased activity[^206^](#_ENREF_206) | |  | | Used to described various haplotypes | | Haplotypes A,B, H1,H2, H7, H8, H9 (PharmGKB.org) | |
| *UGT1A1* |  | | Deficient[^214^](#_ENREF_214)  Decreased activity[^215^](#_ENREF_215) | |  | | An individual carrying two wild type allele/haplotypes | | Wild-type[^215-219^](#_ENREF_215)  Non-carrier[^220-222^](#_ENREF_220)  Homozygous for common allele[^223^](#_ENREF_223) | |
|  |  | |  | |  | | An individual carrying at least one decreased activity allele/haplotypes | | Non-Wild-type[^215^](#_ENREF_215)  Carrier  Heterozygote/homozygote | |
| *CYP3A5* | Normal function | | Expresser[^224^](#_ENREF_224)  Normal Function[^19^](#_ENREF_19)  Wild-type[^225-227^](#_ENREF_225)  Functional[^225^](#_ENREF_225) | |  | | An individual carrying at least one *1 allele | | High expresser[^228^](#_ENREF_228)  Expresser | |
|  | No function | | Lack of activity[^225^](#_ENREF_225)  Loss of activity[^225^](#_ENREF_225)  Reduced expresser[^224^](#_ENREF_224)  Decreased function[^19^](#_ENREF_19) | |  | | An individual carrying no *1 allele | | Low expresser[^228^](#_ENREF_228)  Non-expresser  Reduced metabolizers[^19^](#_ENREF_19) | |

^a^Currently used term(s) in CPIC guidelines.

# Supplemental Figure S1. Terms used to describe allele functional status for drug metabolizing enzymes results by round

Terms in red were eliminated during round listed and not found in the next round

* denotes terms added to the survey for the round listed based on comments made by the experts during the preceding round

The term set in black in round 4 represents the consensus term sets

# Supplemental Figure S2. Terms used to describe allele functional status for non-drug metabolizing enzymes results by round

Terms in red were eliminated during round listed and not found in the next round

* denotes terms added to the survey for the round listed because of comments made by the experts during the preceding round

The term set in black in round 4 represents the consensus term sets

# Supplemental Figure S3. Terms used to describe phenotype for drug metabolizing enzymes results by survey round.

Terms in red were eliminated during round listed and not included in the next round

* denotes terms added to the survey for the round listed because of comments made by the experts during the preceding round

The term set in black in round 4 represents the consensus term sets

# Supplemental Figure S4. Terms used to describe phenotype for non-drug metabolizing enzymes results by survey round.

Terms in red were eliminated during round listed and not included in the next round

* denotes terms added to the survey for the round listed because of comments made by the experts during the preceding round

The term set in black in round 4 represents the consensus term sets

# Supplemental Table S4: LOINC pharmacogenetic interpretation codes

| LOINC | LOINC Component | Answer List |
| --- | --- | --- |
| 50956-2 | HLA-B*57:01 | Positive vs negative |
| 57979-7 | HLA-B*15:02 | Positive vs negative |
| 79711-8 | HLA-B*58:01 | Positive vs negative |
| 79712-6 | HLA-A*31:01 | Positive vs negative |
|  |  |  |
| 79713-4 | TPMT gene product metabolic activity interpretation | Metabolizer status |
| 79714-2 | CYP2C19 gene product metabolic activity interpretation | Metabolizer status |
| 79715-9 | CYP2D6 gene product metabolic activity interpretation | Metabolizer status |
| 79716-7 | CYP2C9 gene product metabolic activity interpretation | Metabolizer status |
| 79717-5 | CYP3A5 gene product metabolic activity interpretation | Metabolizer status |
| 79718-3 | UGT1A1 gene product metabolic activity interpretation | Metabolizer status |
| 79719-1 | DPYD gene product metabolic activity interpretation | Metabolizer status |
| 79720-9 | CYP2B6 gene product metabolic activity interpretation | Metabolizer status |
| 79721-7 | CYP4F2 gene product metabolic activity interpretation | Metabolizer status |
|  |  |  |
| 79722-5 | SLCO1B1 gene product functional interpretation | Functional status |

*See **Supplemental Table S5** for LOINC answer lists for pharmacogenetic interpretation codes

# Supplemental Table S5: LOINC answer lists for pharmacogenetic interpretation codes

| Answer List | Answer ID | Answer (CPIC Phenotype Term) |
| --- | --- | --- |
| Positive vs negative | | |
|  | LA6576-8 | Positive |
|  | LA6577-6 | Negative |
| Metabolizer Status | | |
|  | LA10315-2 | Ultrarapid metabolizer |
|  | LA25390-8 | Rapid metabolizer |
|  | LA25391-6 | Normal metabolizer |
|  | LA10317-8 | Intermediate metabolizer |
|  | LA9657-3 | Poor metabolizer |
| Functional Status | | |
|  | LA25392-4 | Increased function |
|  | LA25393-2 | Normal function |
|  | LA25395-7 | Decreased function |
|  | LA25394-0 | Poor function |

# References

**1.** Tai HL, Fessing MY, Bonten EJ, et al. Enhanced proteasomal degradation of mutant human thiopurine S-methyltransferase (TPMT) in mammalian cells: mechanism for TPMT protein deficiency inherited by TPMT*2, TPMT*3A, TPMT*3B or TPMT*3C. *Pharmacogenetics.* Oct 1999;9(5):641-650.

**2.** McLeod HL, Coulthard S, Thomas AE, et al. Analysis of thiopurine methyltransferase variant alleles in childhood acute lymphoblastic leukaemia. *British journal of haematology.* Jun 1999;105(3):696-700.

**3.** Black AJ, McLeod HL, Capell HA, et al. Thiopurine methyltransferase genotype predicts therapy-limiting severe toxicity from azathioprine. *Annals of internal medicine.* Nov 1 1998;129(9):716-718.

**4.** Hamdan-Khalil R, Allorge D, Lo-Guidice JM, et al. In vitro characterization of four novel non-functional variants of the thiopurine S-methyltransferase. *Biochemical and biophysical research communications.* Oct 3 2003;309(4):1005-1010.

**5.** Sebbag L, Boucher P, Davelu P, et al. Thiopurine S-methyltransferase gene polymorphism is predictive of azathioprine-induced myelosuppression in heart transplant recipients. *Transplantation.* Apr 15 2000;69(7):1524-1527.

**6.** Karas-Kuzelicki N, Jazbec J, Milek M, Mlinaric-Rascan I. Heterozygosity at the TPMT gene locus, augmented by mutated MTHFR gene, predisposes to 6-MP related toxicities in childhood ALL patients. *Leukemia.* May 2009;23(5):971-974.

**7.** Evans WE, Hon YY, Bomgaars L, et al. Preponderance of thiopurine S-methyltransferase deficiency and heterozygosity among patients intolerant to mercaptopurine or azathioprine. *Journal of clinical oncology : official journal of the American Society of Clinical Oncology.* Apr 15 2001;19(8):2293-2301.

**8.** Stocco G, Yang W, Crews KR, et al. PACSIN2 polymorphism influences TPMT activity and mercaptopurine-related gastrointestinal toxicity. *Human molecular genetics.* Nov 1 2012;21(21):4793-4804.

**9.** Ansari A, Hassan C, Duley J, et al. Thiopurine methyltransferase activity and the use of azathioprine in inflammatory bowel disease. *Alimentary pharmacology & therapeutics.* Oct 2002;16(10):1743-1750.

**10.** Van Loon JA, Weinshilboum RM. Thiopurine methyltransferase isozymes in human renal tissue. *Drug metabolism and disposition: the biological fate of chemicals.* Sep-Oct 1990;18(5):632-638.

**11.** Lennard L, Lilleyman JS, Van Loon J, Weinshilboum RM. Genetic variation in response to 6-mercaptopurine for childhood acute lymphoblastic leukaemia. *Lancet.* Jul 28 1990;336(8709):225-229.

**12.** Relling MV, Hancock ML, Rivera GK, et al. Mercaptopurine therapy intolerance and heterozygosity at the thiopurine S-methyltransferase gene locus. *Journal of the National Cancer Institute.* Dec 1 1999;91(23):2001-2008.

**13.** Schwab M, Schaffeler E, Marx C, et al. Azathioprine therapy and adverse drug reactions in patients with inflammatory bowel disease: impact of thiopurine S-methyltransferase polymorphism. *Pharmacogenetics.* Aug 2002;12(6):429-436.

**14.** Kim JH, Cheon JH, Hong SS, et al. Influences of thiopurine methyltransferase genotype and activity on thiopurine-induced leukopenia in Korean patients with inflammatory bowel disease: a retrospective cohort study. *Journal of clinical gastroenterology.* Nov-Dec 2010;44(10):e242-248.

**15.** De Morais SM, Wilkinson GR, Blaisdell J, Meyer UA, Nakamura K, Goldstein JA. Identification of a new genetic defect responsible for the polymorphism of (S)-mephenytoin metabolism in Japanese. *Molecular pharmacology.* Oct 1994;46(4):594-598.

**16.** Ibeanu GC, Goldstein JA, Meyer U, et al. Identification of new human CYP2C19 alleles (CYP2C19*6 and CYP2C19*2B) in a Caucasian poor metabolizer of mephenytoin. *The Journal of pharmacology and experimental therapeutics.* Sep 1998;286(3):1490-1495.

**17.** Rudberg I, Mohebi B, Hermann M, Refsum H, Molden E. Impact of the ultrarapid CYP2C19*17 allele on serum concentration of escitalopram in psychiatric patients. *Clinical pharmacology and therapeutics.* Feb 2008;83(2):322-327.

**18.** Brandt JT, Close SL, Iturria SJ, et al. Common polymorphisms of CYP2C19 and CYP2C9 affect the pharmacokinetic and pharmacodynamic response to clopidogrel but not prasugrel. *Journal of thrombosis and haemostasis : JTH.* Dec 2007;5(12):2429-2436.

**19.** Jeong YH, Kim IS, Park Y, et al. Carriage of cytochrome 2C19 polymorphism is associated with risk of high post-treatment platelet reactivity on high maintenance-dose clopidogrel of 150 mg/day: results of the ACCEL-DOUBLE (Accelerated Platelet Inhibition by a Double Dose of Clopidogrel According to Gene Polymorphism) study. *JACC Cardiovasc Interv.* Jul 2010;3(7):731-741.

**20.** Kim KA, Park PW, Hong SJ, Park JY. The effect of CYP2C19 polymorphism on the pharmacokinetics and pharmacodynamics of clopidogrel: a possible mechanism for clopidogrel resistance. *Clinical pharmacology and therapeutics.* Aug 2008;84(2):236-242.

**21.** Aung AK, Haas DW, Hulgan T, Phillips EJ. Pharmacogenomics of antimicrobial agents. *Pharmacogenomics.* 2014;15(15):1903-1930.

**22.** Mega JL, Close SL, Wiviott SD, et al. Cytochrome P450 genetic polymorphisms and the response to prasugrel: relationship to pharmacokinetic, pharmacodynamic, and clinical outcomes. *Circulation.* May 19 2009;119(19):2553-2560.

**23.** Mega JL, Close SL, Wiviott SD, et al. Cytochrome p-450 polymorphisms and response to clopidogrel. *The New England journal of medicine.* Jan 22 2009;360(4):354-362.

**24.** Simon T, Bhatt DL, Bergougnan L, et al. Genetic polymorphisms and the impact of a higher clopidogrel dose regimen on active metabolite exposure and antiplatelet response in healthy subjects. *Clinical pharmacology and therapeutics.* Aug 2011;90(2):287-295.

**25.** Rideg O, Komocsi A, Magyarlaki T, et al. Impact of genetic variants on post-clopidogrel platelet reactivity in patients after elective percutaneous coronary intervention. *Pharmacogenomics.* Sep 2011;12(9):1269-1280.

**26.** Sorich MJ, Polasek TM, Wiese MD. Systematic review and meta-analysis of the association between cytochrome P450 2C19 genotype and bleeding. *Thromb Haemost.* Jul 2012;108(1):199-200.

**27.** Subraja K, Dkhar SA, Priyadharsini R, et al. Genetic polymorphisms of CYP2C19 influences the response to clopidogrel in ischemic heart disease patients in the South Indian Tamilian population. *European journal of clinical pharmacology.* Mar 2013;69(3):415-422.

**28.** Levin MD, den Hollander JG, van der Holt B, et al. Hepatotoxicity of oral and intravenous voriconazole in relation to cytochrome P450 polymorphisms. *J Antimicrob Chemother.* Nov 2007;60(5):1104-1107.

**29.** Matsumoto K, Ikawa K, Abematsu K, et al. Correlation between voriconazole trough plasma concentration and hepatotoxicity in patients with different CYP2C19 genotypes. *Int J Antimicrob Agents.* Jul 2009;34(1):91-94.

**30.** Varenhorst C, James S, Erlinge D, et al. Genetic variation of CYP2C19 affects both pharmacokinetic and pharmacodynamic responses to clopidogrel but not prasugrel in aspirin-treated patients with coronary artery disease. *European heart journal.* Jul 2009;30(14):1744-1752.

**31.** Gurbel PA, Shuldiner AR, Bliden KP, Ryan K, Pakyz RE, Tantry US. The relation between CYP2C19 genotype and phenotype in stented patients on maintenance dual antiplatelet therapy. *American heart journal.* Mar 2011;161(3):598-604.

**32.** Frere C, Cuisset T, Gaborit B, Alessi MC, Hulot JS. The CYP2C19*17 allele is associated with better platelet response to clopidogrel in patients admitted for non-ST acute coronary syndrome. *Journal of thrombosis and haemostasis : JTH.* Aug 2009;7(8):1409-1411.

**33.** Pare G, Mehta SR, Yusuf S, et al. Effects of CYP2C19 genotype on outcomes of clopidogrel treatment. *The New England journal of medicine.* Oct 28 2010;363(18):1704-1714.

**34.** de Morais SM, Wilkinson GR, Blaisdell J, Nakamura K, Meyer UA, Goldstein JA. The major genetic defect responsible for the polymorphism of S-mephenytoin metabolism in humans. *The Journal of biological chemistry.* Jun 3 1994;269(22):15419-15422.

**35.** Lehr T, Yuan J, Hall D, et al. Integration of absorption, distribution, metabolism, and elimination genotyping data into a population pharmacokinetic analysis of nevirapine. *Pharmacogenetics and genomics.* Nov 2011;21(11):721-730.

**36.** Ferguson RJ, De Morais SM, Benhamou S, et al. A new genetic defect in human CYP2C19: mutation of the initiation codon is responsible for poor metabolism of S-mephenytoin. *The Journal of pharmacology and experimental therapeutics.* Jan 1998;284(1):356-361.

**37.** Ibeanu GC, Blaisdell J, Ghanayem BI, et al. An additional defective allele, CYP2C19*5, contributes to the S-mephenytoin poor metabolizer phenotype in Caucasians. *Pharmacogenetics.* Apr 1998;8(2):129-135.

**38.** Sim SC, Risinger C, Dahl ML, et al. A common novel CYP2C19 gene variant causes ultrarapid drug metabolism relevant for the drug response to proton pump inhibitors and antidepressants. *Clinical pharmacology and therapeutics.* Jan 2006;79(1):103-113.

**39.** Li-Wan-Po A, Girard T, Farndon P, Cooley C, Lithgow J. Pharmacogenetics of CYP2C19: functional and clinical implications of a new variant CYP2C19*17. *British journal of clinical pharmacology.* Mar 2010;69(3):222-230.

**40.** Hulot JS, Collet JP, Silvain J, et al. Cardiovascular risk in clopidogrel-treated patients according to cytochrome P450 2C19*2 loss-of-function allele or proton pump inhibitor coadministration: a systematic meta-analysis. *Journal of the American College of Cardiology.* Jul 6 2010;56(2):134-143.

**41.** Cresci S, Depta JP, Lenzini PA, et al. Cytochrome p450 gene variants, race, and mortality among clopidogrel-treated patients after acute myocardial infarction. *Circ Cardiovasc Genet.* Jun 2014;7(3):277-286.

**42.** Viviani Anselmi C, Briguori C, Roncarati R, et al. Routine assessment of on-clopidogrel platelet reactivity and gene polymorphisms in predicting clinical outcome following drug-eluting stent implantation in patients with stable coronary artery disease. *JACC Cardiovasc Interv.* Nov 2013;6(11):1166-1175.

**43.** Wu H, Qian J, Xu J, et al. Effects of CYP2C19 variant alleles on postclopidogrel platelet reactivity and clinical outcomes in an actual clinical setting in China. *Pharmacogenetics and genomics.* Dec 2012;22(12):887-890.

**44.** Suh JW, Cha MJ, Lee SP, et al. Relationship between statin type and responsiveness to clopidogrel in patients treated with percutaneous coronary intervention: a subgroup analysis of the CILON-T trial. *J Atheroscler Thromb.* 2014;21(2):140-150.

**45.** Boso V, Herrero MJ, Bea S, et al. Increased hospital stay and allograft dysfunction in renal transplant recipients with Cyp2c19 AA variant in SNP rs4244285. *Drug metabolism and disposition: the biological fate of chemicals.* Feb 2013;41(2):480-487.

**46.** Frere C, Cuisset T, Morange PE, et al. Effect of cytochrome p450 polymorphisms on platelet reactivity after treatment with clopidogrel in acute coronary syndrome. *Am J Cardiol.* Apr 15 2008;101(8):1088-1093.

**47.** Gong IY, Crown N, Suen CM, et al. Clarifying the importance of CYP2C19 and PON1 in the mechanism of clopidogrel bioactivation and in vivo antiplatelet response. *European heart journal.* Nov 2012;33(22):2856-2464a.

**48.** Meletiadis J, Chanock S, Walsh TJ. Defining targets for investigating the pharmacogenomics of adverse drug reactions to antifungal agents. *Pharmacogenomics.* May 2008;9(5):561-584.

**49.** Padol S, Yuan Y, Thabane M, Padol IT, Hunt RH. The effect of CYP2C19 polymorphisms on H. pylori eradication rate in dual and triple first-line PPI therapies: a meta-analysis. *Am J Gastroenterol.* Jul 2006;101(7):1467-1475.

**50.** Zhao F, Wang J, Yang Y, et al. Effect of CYP2C19 genetic polymorphisms on the efficacy of proton pump inhibitor-based triple therapy for Helicobacter pylori eradication: a meta-analysis. *Helicobacter.* Dec 2008;13(6):532-541.

**51.** Tang HL, Li Y, Hu YF, Xie HG, Zhai SD. Effects of CYP2C19 loss-of-function variants on the eradication of H. pylori infection in patients treated with proton pump inhibitor-based triple therapy regimens: a meta-analysis of randomized clinical trials. *PLoS One.* 2013;8(4):e62162.

**52.** Scholz I, Oberwittler H, Riedel KD, et al. Pharmacokinetics, metabolism and bioavailability of the triazole antifungal agent voriconazole in relation to CYP2C19 genotype. *British journal of clinical pharmacology.* Dec 2009;68(6):906-915.

**53.** Crettol S, Deglon JJ, Besson J, et al. Methadone enantiomer plasma levels, CYP2B6, CYP2C19, and CYP2C9 genotypes, and response to treatment. *Clinical pharmacology and therapeutics.* Dec 2005;78(6):593-604.

**54.** Peters EJ, Slager SL, Kraft JB, et al. Pharmacokinetic genes do not influence response or tolerance to citalopram in the STAR*D sample. *PLoS One.* 2008;3(4):e1872.

**55.** Umemura K, Furuta T, Kondo K. The common gene variants of CYP2C19 affect pharmacokinetics and pharmacodynamics in an active metabolite of clopidogrel in healthy subjects. *Journal of thrombosis and haemostasis : JTH.* Aug 2008;6(8):1439-1441.

**56.** Jinnai T, Horiuchi H, Makiyama T, et al. Impact of CYP2C19 polymorphisms on the antiplatelet effect of clopidogrel in an actual clinical setting in Japan. *Circ J.* Aug 2009;73(8):1498-1503.

**57.** Hwang SJ, Jeong YH, Kim IS, et al. The cytochrome 2C19*2 and *3 alleles attenuate response to clopidogrel similarly in East Asian patients undergoing elective percutaneous coronary intervention. *Thromb Res.* Jan 2011;127(1):23-28.

**58.** Maeda A, Ando H, Asai T, et al. Differential impacts of CYP2C19 gene polymorphisms on the antiplatelet effects of clopidogrel and ticlopidine. *Clinical pharmacology and therapeutics.* Feb 2011;89(2):229-233.

**59.** Ono T, Kaikita K, Hokimoto S, et al. Determination of cut-off levels for on-clopidogrel platelet aggregation based on functional CYP2C19 gene variants in patients undergoing elective percutaneous coronary intervention. *Thromb Res.* Dec 2011;128(6):e130-136.

**60.** Kim IS, Jeong YH, Park Y, et al. Interaction analysis between genetic polymorphisms and pharmacodynamic effect in patients treated with adjunctive cilostazol to dual antiplatelet therapy: results of the ACCEL-TRIPLE (Accelerated Platelet Inhibition by Triple Antiplatelet Therapy According to Gene Polymorphism) study. *British journal of clinical pharmacology.* Apr 2012;73(4):629-640.

**61.** Sorich MJ, Vitry A, Ward MB, Horowitz JD, McKinnon RA. Prasugrel vs. clopidogrel for cytochrome P450 2C19-genotyped subgroups: integration of the TRITON-TIMI 38 trial data. *Journal of thrombosis and haemostasis : JTH.* Aug 2010;8(8):1678-1684.

**62.** Simon T, Verstuyft C, Mary-Krause M, et al. Genetic determinants of response to clopidogrel and cardiovascular events. *The New England journal of medicine.* Jan 22 2009;360(4):363-375.

**63.** Kelly RP, Close SL, Farid NA, et al. Pharmacokinetics and pharmacodynamics following maintenance doses of prasugrel and clopidogrel in Chinese carriers of CYP2C19 variants. *British journal of clinical pharmacology.* Jan 2012;73(1):93-105.

**64.** Teixeira R, Monteiro P, Marques G, et al. CYP2C19*2 and prognosis after an acute coronary syndrome: Insights from a Portuguese center. *Rev Port Cardiol.* Apr 2012;31(4):265-273.

**65.** Sibbing D, Stegherr J, Latz W, et al. Cytochrome P450 2C19 loss-of-function polymorphism and stent thrombosis following percutaneous coronary intervention. *European heart journal.* Apr 2009;30(8):916-922.

**66.** Sibbing D, Koch W, Gebhard D, et al. Cytochrome 2C19*17 allelic variant, platelet aggregation, bleeding events, and stent thrombosis in clopidogrel-treated patients with coronary stent placement. *Circulation.* Feb 2 2010;121(4):512-518.

**67.** Delaney JT, Ramirez AH, Bowton E, et al. Predicting clopidogrel response using DNA samples linked to an electronic health record. *Clinical pharmacology and therapeutics.* Feb 2012;91(2):257-263.

**68.** Kassimis G, Davlouros P, Xanthopoulou I, Stavrou EF, Athanassiadou A, Alexopoulos D. CYP2C19*2 and other genetic variants affecting platelet response to clopidogrel in patients undergoing percutaneous coronary intervention. *Thromb Res.* Apr 2012;129(4):441-446.

**69.** Kirchheiner J, Klein C, Meineke I, et al. Bupropion and 4-OH-bupropion pharmacokinetics in relation to genetic polymorphisms in CYP2B6. *Pharmacogenetics.* Oct 2003;13(10):619-626.

**70.** Haas DW, Smeaton LM, Shafer RW, et al. Pharmacogenetics of long-term responses to antiretroviral regimens containing Efavirenz and/or Nelfinavir: an Adult Aids Clinical Trials Group Study. *J Infect Dis.* Dec 1 2005;192(11):1931-1942.

**71.** Lee HK, Hu M, Lui S, Ho CS, Wong CK, Tomlinson B. Effects of polymorphisms in ABCG2, SLCO1B1, SLC10A1 and CYP2C9/19 on plasma concentrations of rosuvastatin and lipid response in Chinese patients. *Pharmacogenomics.* Aug 2013;14(11):1283-1294.

**72.** Yu A, Kneller BM, Rettie AE, Haining RL. Expression, purification, biochemical characterization, and comparative function of human cytochrome P450 2D6.1, 2D6.2, 2D6.10, and 2D6.17 allelic isoforms. *The Journal of pharmacology and experimental therapeutics.* Dec 2002;303(3):1291-1300.

**73.** Oscarson M, Hidestrand M, Johansson I, Ingelman-Sundberg M. A combination of mutations in the CYP2D6*17 (CYP2D6Z) allele causes alterations in enzyme function. *Molecular pharmacology.* Dec 1997;52(6):1034-1040.

**74.** Zhang WY, Tu YB, Haining RL, Yu AM. Expression and functional analysis of CYP2D6.24, CYP2D6.26, CYP2D6.27, and CYP2D7 isozymes. *Drug metabolism and disposition: the biological fate of chemicals.* Jan 2009;37(1):1-4.

**75.** Jaquenoud Sirot E, Harenberg S, Vandel P, et al. Multicenter study on the clinical effectiveness, pharmacokinetics, and pharmacogenetics of mirtazapine in depression. *J Clin Psychopharmacol.* Oct 2012;32(5):622-629.

**76.** Voronov P, Przybylo HJ, Jagannathan N. Apnea in a child after oral codeine: a genetic variant - an ultra-rapid metabolizer. *Paediatr Anaesth.* Jul 2007;17(7):684-687.

**77.** Kelly LE, Rieder M, van den Anker J, et al. More codeine fatalities after tonsillectomy in North American children. *Pediatrics.* May 2012;129(5):e1343-1347.

**78.** Sistonen J, Madadi P, Ross CJ, et al. Prediction of codeine toxicity in infants and their mothers using a novel combination of maternal genetic markers. *Clinical pharmacology and therapeutics.* Apr 2012;91(4):692-699.

**79.** Madadi P, Ross CJ, Hayden MR, et al. Pharmacogenetics of neonatal opioid toxicity following maternal use of codeine during breastfeeding: a case-control study. *Clinical pharmacology and therapeutics.* Jan 2009;85(1):31-35.

**80.** Koren G, Cairns J, Chitayat D, Gaedigk A, Leeder SJ. Pharmacogenetics of morphine poisoning in a breastfed neonate of a codeine-prescribed mother. *Lancet.* Aug 19 2006;368(9536):704.

**81.** Ciszkowski C, Madadi P, Phillips MS, Lauwers AE, Koren G. Codeine, ultrarapid-metabolism genotype, and postoperative death. *The New England journal of medicine.* Aug 20 2009;361(8):827-828.

**82.** VanderVaart S, Berger H, Sistonen J, et al. CYP2D6 polymorphisms and codeine analgesia in postpartum pain management: a pilot study. *Ther Drug Monit.* Aug 2011;33(4):425-432.

**83.** Gasche Y, Daali Y, Fathi M, et al. Codeine intoxication associated with ultrarapid CYP2D6 metabolism. *The New England journal of medicine.* Dec 30 2004;351(27):2827-2831.

**84.** Foster A, Mobley E, Wang Z. Complicated pain management in a CYP450 2D6 poor metabolizer. *Pain Pract.* Dec 2007;7(4):352-356.

**85.** Vevelstad M, Pettersen S, Tallaksen C, Brors O. O-demethylation of codeine to morphine inhibited by low-dose levomepromazine. *European journal of clinical pharmacology.* Aug 2009;65(8):795-801.

**86.** Lotsch J, Skarke C, Schmidt H, et al. Evidence for morphine-independent central nervous opioid effects after administration of codeine: contribution of other codeine metabolites. *Clinical pharmacology and therapeutics.* Jan 2006;79(1):35-48.

**87.** Fagerlund TH, Braaten O. No pain relief from codeine...? An introduction to pharmacogenomics. *Acta Anaesthesiol Scand.* Feb 2001;45(2):140-149.

**88.** Kirchheiner J, Schmidt H, Tzvetkov M, et al. Pharmacokinetics of codeine and its metabolite morphine in ultra-rapid metabolizers due to CYP2D6 duplication. *The pharmacogenomics journal.* Aug 2007;7(4):257-265.

**89.** Shen H, He MM, Liu H, et al. Comparative metabolic capabilities and inhibitory profiles of CYP2D6.1, CYP2D6.10, and CYP2D6.17. *Drug metabolism and disposition: the biological fate of chemicals.* Aug 2007;35(8):1292-1300.

**90.** Sindrup SH, Brosen K, Bjerring P, et al. Codeine increases pain thresholds to copper vapor laser stimuli in extensive but not poor metabolizers of sparteine. *Clinical pharmacology and therapeutics.* Dec 1990;48(6):686-693.

**91.** Desmeules J, Gascon MP, Dayer P, Magistris M. Impact of environmental and genetic factors on codeine analgesia. *European journal of clinical pharmacology.* 1991;41(1):23-26.

**92.** Persson K, Sjostrom S, Sigurdardottir I, Molnar V, Hammarlund-Udenaes M, Rane A. Patient-controlled analgesia (PCA) with codeine for postoperative pain relief in ten extensive metabolisers and one poor metaboliser of dextromethorphan. *British journal of clinical pharmacology.* Feb 1995;39(2):182-186.

**93.** Offer SM, Wegner NJ, Fossum C, Wang K, Diasio RB. Phenotypic profiling of DPYD variations relevant to 5-fluorouracil sensitivity using real-time cellular analysis and in vitro measurement of enzyme activity. *Cancer research.* Mar 15 2013;73(6):1958-1968.

**94.** Sulzyc-Bielicka V, Binczak-Kuleta A, Pioch W, et al. 5-Fluorouracil toxicity-attributable IVS14 + 1G > A mutation of the dihydropyrimidine dehydrogenase gene in Polish colorectal cancer patients. *Pharmacological reports : PR.* Mar-Apr 2008;60(2):238-242.

**95.** van Kuilenburg AB, Haasjes J, Richel DJ, et al. Clinical implications of dihydropyrimidine dehydrogenase (DPD) deficiency in patients with severe 5-fluorouracil-associated toxicity: identification of new mutations in the DPD gene. *Clinical cancer research : an official journal of the American Association for Cancer Research.* Dec 2000;6(12):4705-4712.

**96.** Johnson MR, Wang K, Diasio RB. Profound dihydropyrimidine dehydrogenase deficiency resulting from a novel compound heterozygote genotype. *Clinical cancer research : an official journal of the American Association for Cancer Research.* Mar 2002;8(3):768-774.

**97.** Salgueiro N, Veiga I, Fragoso M, et al. Mutations in exon 14 of dihydropyrimidine dehydrogenase and 5-Fluorouracil toxicity in Portuguese colorectal cancer patients. *Genetics in medicine : official journal of the American College of Medical Genetics.* Mar-Apr 2004;6(2):102-107.

**98.** Maring JG, van Kuilenburg AB, Haasjes J, et al. Reduced 5-FU clearance in a patient with low DPD activity due to heterozygosity for a mutant allele of the DPYD gene. *British journal of cancer.* Apr 8 2002;86(7):1028-1033.

**99.** Van Kuilenburg AB, Meinsma R, Zoetekouw L, Van Gennip AH. High prevalence of the IVS14 + 1G>A mutation in the dihydropyrimidine dehydrogenase gene of patients with severe 5-fluorouracil-associated toxicity. *Pharmacogenetics.* Oct 2002;12(7):555-558.

**100.** Collie-Duguid ES, Etienne MC, Milano G, McLeod HL. Known variant DPYD alleles do not explain DPD deficiency in cancer patients. *Pharmacogenetics.* Apr 2000;10(3):217-223.

**101.** Boisdron-Celle M, Remaud G, Traore S, et al. 5-Fluorouracil-related severe toxicity: a comparison of different methods for the pretherapeutic detection of dihydropyrimidine dehydrogenase deficiency. *Cancer letters.* May 8 2007;249(2):271-282.

**102.** Morel A, Boisdron-Celle M, Fey L, et al. Clinical relevance of different dihydropyrimidine dehydrogenase gene single nucleotide polymorphisms on 5-fluorouracil tolerance. *Molecular cancer therapeutics.* Nov 2006;5(11):2895-2904.

**103.** Loganayagam A, Arenas Hernandez M, Corrigan A, et al. Pharmacogenetic variants in the DPYD, TYMS, CDA and MTHFR genes are clinically significant predictors of fluoropyrimidine toxicity. *British journal of cancer.* Jun 25 2013;108(12):2505-2515.

**104.** Offer SM, Lee AM, Mattison LK, Fossum C, Wegner NJ, Diasio RB. A DPYD variant (Y186C) in individuals of african ancestry is associated with reduced DPD enzyme activity. *Clinical pharmacology and therapeutics.* Jul 2013;94(1):158-166.

**105.** Saif MW, Ezzeldin H, Vance K, Sellers S, Diasio RB. DPYD*2A mutation: the most common mutation associated with DPD deficiency. *Cancer chemotherapy and pharmacology.* Sep 2007;60(4):503-507.

**106.** Capitain O, Boisdron-Celle M, Poirier AL, Abadie-Lacourtoisie S, Morel A, Gamelin E. The influence of fluorouracil outcome parameters on tolerance and efficacy in patients with advanced colorectal cancer. *The pharmacogenomics journal.* Aug 2008;8(4):256-267.

**107.** Ho RH, Choi L, Lee W, et al. Effect of drug transporter genotypes on pravastatin disposition in European- and African-American participants. *Pharmacogenetics and genomics.* Aug 2007;17(8):647-656.

**108.** Carr DF, O'Meara H, Jorgensen AL, et al. SLCO1B1 genetic variant associated with statin-induced myopathy: a proof-of-concept study using the clinical practice research datalink. *Clinical pharmacology and therapeutics.* Dec 2013;94(6):695-701.

**109.** Danik JS, Chasman DI, MacFadyen JG, Nyberg F, Barratt BJ, Ridker PM. Lack of association between SLCO1B1 polymorphisms and clinical myalgia following rosuvastatin therapy. *American heart journal.* Jun 2013;165(6):1008-1014.

**110.** Brunham LR, Lansberg PJ, Zhang L, et al. Differential effect of the rs4149056 variant in SLCO1B1 on myopathy associated with simvastatin and atorvastatin. *The pharmacogenomics journal.* Jun 2012;12(3):233-237.

**111.** Santos PC, Gagliardi AC, Miname MH, et al. SLCO1B1 haplotypes are not associated with atorvastatin-induced myalgia in Brazilian patients with familial hypercholesterolemia. *European journal of clinical pharmacology.* Mar 2012;68(3):273-279.

**112.** Akao H, Polisecki E, Kajinami K, et al. Genetic variation at the SLCO1B1 gene locus and low density lipoprotein cholesterol lowering response to pravastatin in the elderly. *Atherosclerosis.* Feb 2012;220(2):413-417.

**113.** Bailey KM, Romaine SP, Jackson BM, et al. Hepatic metabolism and transporter gene variants enhance response to rosuvastatin in patients with acute myocardial infarction: the GEOSTAT-1 Study. *Circ Cardiovasc Genet.* Jun 2010;3(3):276-285.

**114.** Yang GP, Yuan H, Tang B, et al. Lack of effect of genetic polymorphisms of SLCO1B1 on the lipid-lowering response to pitavastatin in Chinese patients. *Acta Pharmacol Sin.* Mar 2010;31(3):382-386.

**115.** Pasanen MK, Neuvonen M, Neuvonen PJ, Niemi M. SLCO1B1 polymorphism markedly affects the pharmacokinetics of simvastatin acid. *Pharmacogenetics and genomics.* Dec 2006;16(12):873-879.

**116.** Takane H, Miyata M, Burioka N, et al. Pharmacogenetic determinants of variability in lipid-lowering response to pravastatin therapy. *J Hum Genet.* 2006;51(9):822-826.

**117.** Kameyama Y, Yamashita K, Kobayashi K, Hosokawa M, Chiba K. Functional characterization of SLCO1B1 (OATP-C) variants, SLCO1B1*5, SLCO1B1*15 and SLCO1B1*15+C1007G, by using transient expression systems of HeLa and HEK293 cells. *Pharmacogenetics and genomics.* Jul 2005;15(7):513-522.

**118.** Nozawa T, Nakajima M, Tamai I, et al. Genetic polymorphisms of human organic anion transporters OATP-C (SLC21A6) and OATP-B (SLC21A9): allele frequencies in the Japanese population and functional analysis. *The Journal of pharmacology and experimental therapeutics.* Aug 2002;302(2):804-813.

**119.** Ho RH, Tirona RG, Leake BF, et al. Drug and bile acid transporters in rosuvastatin hepatic uptake: function, expression, and pharmacogenetics. *Gastroenterology.* May 2006;130(6):1793-1806.

**120.** Iwai M, Suzuki H, Ieiri I, Otsubo K, Sugiyama Y. Functional analysis of single nucleotide polymorphisms of hepatic organic anion transporter OATP1B1 (OATP-C). *Pharmacogenetics.* Nov 2004;14(11):749-757.

**121.** Voora D, Shah SH, Spasojevic I, et al. The SLCO1B1*5 genetic variant is associated with statin-induced side effects. *Journal of the American College of Cardiology.* Oct 20 2009;54(17):1609-1616.

**122.** Zhang W, Chen BL, Ozdemir V, et al. SLCO1B1 521T-->C functional genetic polymorphism and lipid-lowering efficacy of multiple-dose pravastatin in Chinese coronary heart disease patients. *British journal of clinical pharmacology.* Sep 2007;64(3):346-352.

**123.** He J, Qiu Z, Li N, et al. Effects of SLCO1B1 polymorphisms on the pharmacokinetics and pharmacodynamics of repaglinide in healthy Chinese volunteers. *European journal of clinical pharmacology.* Jul 2011;67(7):701-707.

**124.** Kwara A, Cao L, Yang H, et al. Factors associated with variability in rifampin plasma pharmacokinetics and the relationship between rifampin concentrations and induction of efavirenz clearance. *Pharmacotherapy.* Mar 2014;34(3):265-271.

**125.** Swart M, Skelton M, Ren Y, Smith P, Takuva S, Dandara C. High predictive value of CYP2B6 SNPs for steady-state plasma efavirenz levels in South African HIV/AIDS patients. *Pharmacogenetics and genomics.* Aug 2013;23(8):415-427.

**126.** Rocha V, Porcher R, Fernandes JF, et al. Association of drug metabolism gene polymorphisms with toxicities, graft-versus-host disease and survival after HLA-identical sibling hematopoietic stem cell transplantation for patients with leukemia. *Leukemia.* Mar 2009;23(3):545-556.

**127.** Tsuchiya K, Gatanaga H, Tachikawa N, et al. Homozygous CYP2B6 *6 (Q172H and K262R) correlates with high plasma efavirenz concentrations in HIV-1 patients treated with standard efavirenz-containing regimens. *Biochemical and biophysical research communications.* Jul 9 2004;319(4):1322-1326.

**128.** Desta Z, Saussele T, Ward B, et al. Impact of CYP2B6 polymorphism on hepatic efavirenz metabolism in vitro. *Pharmacogenomics.* Jun 2007;8(6):547-558.

**129.** Eap CB, Crettol S, Rougier JS, et al. Stereoselective block of hERG channel by (S)-methadone and QT interval prolongation in CYP2B6 slow metabolizers. *Clinical pharmacology and therapeutics.* May 2007;81(5):719-728.

**130.** Leger P, Dillingham R, Beauharnais CA, et al. CYP2B6 variants and plasma efavirenz concentrations during antiretroviral therapy in Port-au-Prince, Haiti. *J Infect Dis.* Sep 15 2009;200(6):955-964.

**131.** Mukonzo JK, Nanzigu S, Waako P, Ogwal-Okeng J, Gustafson LL, Aklillu E. CYP2B6 genotype, but not rifampicin-based anti-TB cotreatments, explains variability in long-term efavirenz plasma exposure. *Pharmacogenomics.* Aug 2014;15(11):1423-1435.

**132.** Haas DW, Severe P, Jean Juste MA, Pape JW, Fitzgerald DW. Functional CYP2B6 variants and virologic response to an efavirenz-containing regimen in Port-au-Prince, Haiti. *J Antimicrob Chemother.* Aug 2014;69(8):2187-2190.

**133.** Uppugunduri CR, Rezgui MA, Diaz PH, et al. The association of cytochrome P450 genetic polymorphisms with sulfolane formation and the efficacy of a busulfan-based conditioning regimen in pediatric patients undergoing hematopoietic stem cell transplantation. *The pharmacogenomics journal.* Jun 2014;14(3):263-271.

**134.** Xu C, Quinney SK, Guo Y, Hall SD, Li L, Desta Z. CYP2B6 pharmacogenetics-based in vitro-in vivo extrapolation of efavirenz clearance by physiologically based pharmacokinetic modeling. *Drug metabolism and disposition: the biological fate of chemicals.* Dec 2013;41(12):2004-2011.

**135.** Bray J, Sludden J, Griffin MJ, et al. Influence of pharmacogenetics on response and toxicity in breast cancer patients treated with doxorubicin and cyclophosphamide. *British journal of cancer.* Mar 16 2010;102(6):1003-1009.

**136.** Sukasem C, Chamnanphon M, Koomdee N, et al. High plasma efavirenz concentration and CYP2B6 polymorphisms in Thai HIV-1 infections. *Drug Metab Pharmacokinet.* 2013;28(5):391-397.

**137.** Zhu AZ, Cox LS, Nollen N, et al. CYP2B6 and bupropion's smoking-cessation pharmacology: the role of hydroxybupropion. *Clinical pharmacology and therapeutics.* Dec 2012;92(6):771-777.

**138.** Abdelhady AM, Desta Z, Jiang F, Yeo CW, Shin JG, Overholser BR. Population pharmacogenetic-based pharmacokinetic modeling of efavirenz, 7-hydroxy- and 8-hydroxyefavirenz. *J Clin Pharmacol.* Jan 2014;54(1):87-96.

**139.** Uttayamakul S, Likanonsakul S, Manosuthi W, et al. Effects of CYP2B6 G516T polymorphisms on plasma efavirenz and nevirapine levels when co-administered with rifampicin in HIV/TB co-infected Thai adults. *AIDS Res Ther.* 2010;7:8.

**140.** Elens L, Vandercam B, Yombi JC, Lison D, Wallemacq P, Haufroid V. Influence of host genetic factors on efavirenz plasma and intracellular pharmacokinetics in HIV-1-infected patients. *Pharmacogenomics.* Sep 2010;11(9):1223-1234.

**141.** Yimer G, Amogne W, Habtewold A, et al. High plasma efavirenz level and CYP2B6*6 are associated with efavirenz-based HAART-induced liver injury in the treatment of naive HIV patients from Ethiopia: a prospective cohort study. *The pharmacogenomics journal.* Dec 2012;12(6):499-506.

**142.** Ngaimisi E, Mugusi S, Minzi O, et al. Effect of rifampicin and CYP2B6 genotype on long-term efavirenz autoinduction and plasma exposure in HIV patients with or without tuberculosis. *Clinical pharmacology and therapeutics.* Sep 2011;90(3):406-413.

**143.** Manosuthi W, Sukasem C, Thongyen S, Nilkamhang S, Manosuthi S, Sungkanuparph S. CYP2B6 18492T->C polymorphism compromises efavirenz concentration in coinfected HIV and tuberculosis patients carrying CYP2B6 haplotype *1/*1. *Antimicrobial agents and chemotherapy.* 2014;58(4):2268-2273.

**144.** Shinde DD, Kim HS, Choi JS, et al. Different effects of clopidogrel and clarithromycin on the enantioselective pharmacokinetics of sibutramine and its active metabolites in healthy subjects. *J Clin Pharmacol.* May 2013;53(5):550-558.

**145.** Johnstone E, Benowitz N, Cargill A, et al. Determinants of the rate of nicotine metabolism and effects on smoking behavior. *Clinical pharmacology and therapeutics.* Oct 2006;80(4):319-330.

**146.** Rotger M, Tegude H, Colombo S, et al. Predictive value of known and novel alleles of CYP2B6 for efavirenz plasma concentrations in HIV-infected individuals. *Clinical pharmacology and therapeutics.* Apr 2007;81(4):557-566.

**147.** Zukunft J, Lang T, Richter T, et al. A natural CYP2B6 TATA box polymorphism (-82T--> C) leading to enhanced transcription and relocation of the transcriptional start site. *Molecular pharmacology.* May 2005;67(5):1772-1782.

**148.** Jamshidi Y, Moreton M, McKeown DA, et al. Tribal ethnicity and CYP2B6 genetics in Ugandan and Zimbabwean populations in the UK: implications for efavirenz dosing in HIV infection. *J Antimicrob Chemother.* Dec 2010;65(12):2614-2619.

**149.** Wang J, Sonnerborg A, Rane A, et al. Identification of a novel specific CYP2B6 allele in Africans causing impaired metabolism of the HIV drug efavirenz. *Pharmacogenetics and genomics.* Mar 2006;16(3):191-198.

**150.** Zhang H, Sridar C, Kenaan C, Amunugama H, Ballou DP, Hollenberg PF. Polymorphic variants of cytochrome P450 2B6 (CYP2B6.4-CYP2B6.9) exhibit altered rates of metabolism for bupropion and efavirenz: a charge-reversal mutation in the K139E variant (CYP2B6.8) impairs formation of a functional cytochrome p450-reductase complex. *The Journal of pharmacology and experimental therapeutics.* Sep 2011;338(3):803-809.

**151.** Sukasem C, Chamnanphon M, Koomdee N, et al. Pharmacogenetics and clinical biomarkers for subtherapeutic plasma efavirenz concentration in HIV-1 infected Thai adults. *Drug Metab Pharmacokinet.* 2014;29(4):289-295.

**152.** Manosuthi W, Sukasem C, Lueangniyomkul A, et al. Impact of pharmacogenetic markers of CYP2B6, clinical factors, and drug-drug interaction on efavirenz concentrations in HIV/tuberculosis-coinfected patients. *Antimicrobial agents and chemotherapy.* Feb 2013;57(2):1019-1024.

**153.** Ciccacci C, Di Fusco D, Marazzi MC, et al. Association between CYP2B6 polymorphisms and Nevirapine-induced SJS/TEN: a pharmacogenetics study. *European journal of clinical pharmacology.* Nov 2013;69(11):1909-1916.

**154.** Hung CC, Chiou MH, Huang BH, et al. Impact of genetic polymorphisms in ABCB1, CYP2B6, OPRM1, ANKK1 and DRD2 genes on methadone therapy in Han Chinese patients. *Pharmacogenomics.* Nov 2011;12(11):1525-1533.

**155.** Kwara A, Lartey M, Sagoe KW, Rzek NL, Court MH. CYP2B6 (c.516G-->T) and CYP2A6 (*9B and/or *17) polymorphisms are independent predictors of efavirenz plasma concentrations in HIV-infected patients. *British journal of clinical pharmacology.* Apr 2009;67(4):427-436.

**156.** Viljoen M, Karlsson MO, Meyers TM, Gous H, Dandara C, Rheeders M. Influence of CYP2B6 516G>T polymorphism and interoccasion variability (IOV) on the population pharmacokinetics of efavirenz in HIV-infected South African children. *European journal of clinical pharmacology.* Apr 2012;68(4):339-347.

**157.** Heil SG, van der Ende ME, Schenk PW, et al. Associations between ABCB1, CYP2A6, CYP2B6, CYP2D6, and CYP3A5 alleles in relation to efavirenz and nevirapine pharmacokinetics in HIV-infected individuals. *Ther Drug Monit.* Apr 2012;34(2):153-159.

**158.** Holzinger ER, Grady B, Ritchie MD, et al. Genome-wide association study of plasma efavirenz pharmacokinetics in AIDS Clinical Trials Group protocols implicates several CYP2B6 variants. *Pharmacogenetics and genomics.* Dec 2012;22(12):858-867.

**159.** Cortes CP, Siccardi M, Chaikan A, Owen A, Zhang G, la Porte CJ. Correlates of efavirenz exposure in Chilean patients affected with human immunodeficiency virus reveals a novel association with a polymorphism in the constitutive androstane receptor. *Ther Drug Monit.* Feb 2013;35(1):78-83.

**160.** Sanchez Martin A, Cabrera Figueroa S, Cruz Guerrero R, Hurtado LP, Hurle AD, Carracedo Alvarez A. Impact of pharmacogenetics on CNS side effects related to efavirenz. *Pharmacogenomics.* Jul 2013;14(10):1167-1178.

**161.** Burger D, van der Heiden I, la Porte C, et al. Interpatient variability in the pharmacokinetics of the HIV non-nucleoside reverse transcriptase inhibitor efavirenz: the effect of gender, race, and CYP2B6 polymorphism. *British journal of clinical pharmacology.* Feb 2006;61(2):148-154.

**162.** Mukonzo JK, Owen JS, Ogwal-Okeng J, et al. Pharmacogenetic-based efavirenz dose modification: suggestions for an African population and the different CYP2B6 genotypes. *PLoS One.* 2014;9(1):e86919.

**163.** Mukonzo JK, Okwera A, Nakasujja N, et al. Influence of efavirenz pharmacokinetics and pharmacogenetics on neuropsychological disorders in Ugandan HIV-positive patients with or without tuberculosis: a prospective cohort study. *BMC Infect Dis.* 2013;13:261.

**164.** Ciccacci C, Borgiani P, Ceffa S, et al. Nevirapine-induced hepatotoxicity and pharmacogenetics: a retrospective study in a population from Mozambique. *Pharmacogenomics.* Jan 2010;11(1):23-31.

**165.** Gounden V, van Niekerk C, Snyman T, George JA. Presence of the CYP2B6 516G> T polymorphism, increased plasma Efavirenz concentrations and early neuropsychiatric side effects in South African HIV-infected patients. *AIDS Res Ther.* 2010;7:32.

**166.** Coelho AV, Silva SP, de Alencar LC, et al. ABCB1 and ABCC1 variants associated with virological failure of first-line protease inhibitors antiretroviral regimens in Northeast Brazil patients. *J Clin Pharmacol.* Dec 2013;53(12):1286-1293.

**167.** Schipani A, Wyen C, Mahungu T, et al. Integration of population pharmacokinetics and pharmacogenetics: an aid to optimal nevirapine dose selection in HIV-infected individuals. *J Antimicrob Chemother.* Jun 2011;66(6):1332-1339.

**168.** Yuan J, Guo S, Hall D, et al. Toxicogenomics of nevirapine-associated cutaneous and hepatic adverse events among populations of African, Asian, and European descent. *AIDS.* Jun 19 2011;25(10):1271-1280.

**169.** Gozalo C, Gerard L, Loiseau P, et al. Pharmacogenetics of toxicity, plasma trough concentration and treatment outcome with nevirapine-containing regimen in anti-retroviral-naive HIV-infected adults: an exploratory study of the TRIANON ANRS 081 trial. *Basic Clin Pharmacol Toxicol.* Dec 2011;109(6):513-520.

**170.** Lehmann DS, Ribaudo HJ, Daar ES, et al. Genome-wide association study of virologic response with efavirenz-containing or abacavir-containing regimens in AIDS clinical trials group protocols. *Pharmacogenetics and genomics.* Feb 2015;25(2):51-59.

**171.** Carr DF, Chaponda M, Cornejo Castro EM, et al. CYP2B6 c.983T>C polymorphism is associated with nevirapine hypersensitivity in Malawian and Ugandan HIV populations. *J Antimicrob Chemother.* Dec 2014;69(12):3329-3334.

**172.** Dickinson L, Chaponda M, Carr DF, et al. Population pharmacokinetic and pharmacogenetic analysis of nevirapine in hypersensitive and tolerant HIV-infected patients from Malawi. *Antimicrobial agents and chemotherapy.* 2014;58(2):706-712.

**173.** Lerman C, Shields PG, Wileyto EP, et al. Pharmacogenetic investigation of smoking cessation treatment. *Pharmacogenetics.* Nov 2002;12(8):627-634.

**174.** Levran O, Peles E, Hamon S, Randesi M, Adelson M, Kreek MJ. CYP2B6 SNPs are associated with methadone dose required for effective treatment of opioid addiction. *Addict Biol.* Jul 2013;18(4):709-716.

**175.** Sukasem C, Cressey TR, Prapaithong P, et al. Pharmacogenetic markers of CYP2B6 associated with efavirenz plasma concentrations in HIV-1 infected Thai adults. *British journal of clinical pharmacology.* Dec 2012;74(6):1005-1012.

**176.** Loryan I, Lindqvist M, Johansson I, et al. Influence of sex on propofol metabolism, a pilot study: implications for propofol anesthesia. *European journal of clinical pharmacology.* Apr 2012;68(4):397-406.

**177.** Calcagno A, D'Avolio A, Simiele M, et al. Influence of CYP2B6 and ABCB1 SNPs on nevirapine plasma concentrations in Burundese HIV-positive patients using dried sample spot devices. *British journal of clinical pharmacology.* Jul 2012;74(1):134-140.

**178.** Benowitz NL, Zhu AZ, Tyndale RF, Dempsey D, Jacob P, 3rd. Influence of CYP2B6 genetic variants on plasma and urine concentrations of bupropion and metabolites at steady state. *Pharmacogenetics and genomics.* Mar 2013;23(3):135-141.

**179.** Klein K, Lang T, Saussele T, et al. Genetic variability of CYP2B6 in populations of African and Asian origin: allele frequencies, novel functional variants, and possible implications for anti-HIV therapy with efavirenz. *Pharmacogenetics and genomics.* Dec 2005;15(12):861-873.

**180.** Wyen C, Hendra H, Vogel M, et al. Impact of CYP2B6 983T>C polymorphism on non-nucleoside reverse transcriptase inhibitor plasma concentrations in HIV-infected patients. *J Antimicrob Chemother.* Apr 2008;61(4):914-918.

**181.** Martin AS, Gomez AI, Garcia-Berrocal B, et al. Dose reduction of efavirenz: an observational study describing cost-effectiveness, pharmacokinetics and pharmacogenetics. *Pharmacogenomics.* May 2014;15(7):997-1006.

**182.** Kassogue Y, Quachouh M, Dehbi H, Quessar A, Benchekroun S, Nadifi S. Functional polymorphism of CYP2B6 G15631T is associated with hematologic and cytogenetic response in chronic myeloid leukemia patients treated with imatinib. *Med Oncol.* Jan 2014;31(1):782.

**183.** Kwara A, Lartey M, Sagoe KW, Kenu E, Court MH. CYP2B6, CYP2A6 and UGT2B7 genetic polymorphisms are predictors of efavirenz mid-dose concentration in HIV-infected patients. *AIDS.* Oct 23 2009;23(16):2101-2106.

**184.** David SP, Brown RA, Papandonatos GD, et al. Pharmacogenetic clinical trial of sustained-release bupropion for smoking cessation. *Nicotine Tob Res.* Aug 2007;9(8):821-833.

**185.** Wyen C, Hendra H, Siccardi M, et al. Cytochrome P450 2B6 (CYP2B6) and constitutive androstane receptor (CAR) polymorphisms are associated with early discontinuation of efavirenz-containing regimens. *J Antimicrob Chemother.* Sep 2011;66(9):2092-2098.

**186.** Haas DW, Kwara A, Richardson DM, et al. Secondary metabolism pathway polymorphisms and plasma efavirenz concentrations in HIV-infected adults with CYP2B6 slow metabolizer genotypes. *J Antimicrob Chemother.* Aug 2014;69(8):2175-2182.

**187.** Bienvenu E, Swart M, Dandara C, Ashton M. The role of genetic polymorphisms in cytochrome P450 and effects of tuberculosis co-treatment on the predictive value of CYP2B6 SNPs and on efavirenz plasma levels in adult HIV patients. *Antiviral Res.* Feb 2014;102:44-53.

**188.** Gandhi M, Greenblatt RM, Bacchetti P, et al. A single-nucleotide polymorphism in CYP2B6 leads to >3-fold increases in efavirenz concentrations in plasma and hair among HIV-infected women. *J Infect Dis.* Nov 2012;206(9):1453-1461.

**189.** Habtewold A, Amogne W, Makonnen E, et al. Pharmacogenetic and pharmacokinetic aspects of CYP3A induction by efavirenz in HIV patients. *The pharmacogenomics journal.* Dec 2013;13(6):484-489.

**190.** Lacut K, Ayme-Dietrich E, Gourhant L, et al. Impact of genetic factors (VKORC1, CYP2C9, CYP4F2 and EPHX1) on the anticoagulation response to fluindione. *British journal of clinical pharmacology.* Mar 2012;73(3):428-436.

**191.** Smires FZ, Moreau C, Habbal R, et al. Influence of genetics and non-genetic factors on acenocoumarol maintenance dose requirement in Moroccan patients. *J Clin Pharm Ther.* Oct 2012;37(5):594-598.

**192.** Moreau C, Bajolle F, Siguret V, et al. Vitamin K antagonists in children with heart disease: height and VKORC1 genotype are the main determinants of the warfarin dose requirement. *Blood.* Jan 19 2012;119(3):861-867.

**193.** Hummers-Pradier E, Hess S, Adham IM, Papke T, Pieske B, Kochen MM. Determination of bleeding risk using genetic markers in patients taking phenprocoumon. *European journal of clinical pharmacology.* Jul 2003;59(3):213-219.

**194.** Wadelius M, Chen LY, Downes K, et al. Common VKORC1 and GGCX polymorphisms associated with warfarin dose. *The pharmacogenomics journal.* 2005;5(4):262-270.

**195.** Argikar UA, Cloyd JC, Birnbaum AK, et al. Paradoxical urinary phenytoin metabolite (S)/(R) ratios in CYP2C19*1/*2 patients. *Epilepsy Res.* Sep 2006;71(1):54-63.

**196.** Lee SY, Kim JS, Kim JW. A case of intolerance to warfarin dosing in an intermediate metabolizer of CYP2C9. *Yonsei Med J.* Dec 31 2005;46(6):843-846.

**197.** Scott SA, Edelmann L, Kornreich R, Erazo M, Desnick RJ. CYP2C9, CYP2C19 and CYP2D6 allele frequencies in the Ashkenazi Jewish population. *Pharmacogenomics.* Jul 2007;8(7):721-730.

**198.** Ozer M, Demirci Y, Hizel C, et al. Impact of genetic factors (CYP2C9, VKORC1 and CYP4F2) on warfarin dose requirement in the Turkish population. *Basic Clin Pharmacol Toxicol.* Mar 2013;112(3):209-214.

**199.** Kesavan R, Narayan SK, Adithan C. Influence of CYP2C9 and CYP2C19 genetic polymorphisms on phenytoin-induced neurological toxicity in Indian epileptic patients. *European journal of clinical pharmacology.* Jul 2010;66(7):689-696.

**200.** Carlquist JF, Horne BD, Muhlestein JB, et al. Genotypes of the cytochrome p450 isoform, CYP2C9, and the vitamin K epoxide reductase complex subunit 1 conjointly determine stable warfarin dose: a prospective study. *J Thromb Thrombolysis.* Dec 2006;22(3):191-197.

**201.** Hamberg AK, Wadelius M, Friberg LE, Biss TT, Kamali F, Jonsson EN. Characterizing variability in warfarin dose requirements in children using modelling and simulation. *British journal of clinical pharmacology.* Jul 2014;78(1):158-169.

**202.** Ramasamy K, Narayan SK, Shewade DG, Chandrasekaran A. Influence of CYP2C9 genetic polymorphism and undernourishment on plasma-free phenytoin concentrations in epileptic patients. *Ther Drug Monit.* Dec 2010;32(6):762-766.

**203.** Phabphal K, Geater A, Limapichat K, Sathirapanya P, Setthawatcharawanich S, Leelawattana R. The association between CYP 2C9 polymorphism and bone health. *Seizure.* Nov 2013;22(9):766-771.

**204.** Kirchheiner J, Brockmoller J. Clinical consequences of cytochrome P450 2C9 polymorphisms. *Clinical pharmacology and therapeutics.* Jan 2005;77(1):1-16.

**205.** Mamiya K, Ieiri I, Shimamoto J, et al. The effects of genetic polymorphisms of CYP2C9 and CYP2C19 on phenytoin metabolism in Japanese adult patients with epilepsy: studies in stereoselective hydroxylation and population pharmacokinetics. *Epilepsia.* Dec 1998;39(12):1317-1323.

**206.** Cavallari LH, Langaee TY, Momary KM, et al. Genetic and clinical predictors of warfarin dose requirements in African Americans. *Clinical pharmacology and therapeutics.* Apr 2010;87(4):459-464.

**207.** Singh O, Sandanaraj E, Subramanian K, Lee LH, Chowbay B. Influence of CYP4F2 rs2108622 (V433M) on warfarin dose requirement in Asian patients. *Drug Metab Pharmacokinet.* 2011;26(2):130-136.

**208.** Lubitz SA, Scott SA, Rothlauf EB, et al. Comparative performance of gene-based warfarin dosing algorithms in a multiethnic population. *Journal of thrombosis and haemostasis : JTH.* May 2010;8(5):1018-1026.

**209.** Geisen C, Luxembourg B, Watzka M, et al. Prediction of phenprocoumon maintenance dose and phenprocoumon plasma concentration by genetic and non-genetic parameters. *European journal of clinical pharmacology.* Apr 2011;67(4):371-381.

**210.** Roustit M, Fonrose X, Montani D, et al. CYP2C9, SLCO1B1, SLCO1B3, and ABCB11 polymorphisms in patients with bosentan-induced liver toxicity. *Clinical pharmacology and therapeutics.* Jun 2014;95(6):583-585.

**211.** Caraco Y, Muszkat M, Wood AJ. Phenytoin metabolic ratio: a putative marker of CYP2C9 activity in vivo. *Pharmacogenetics.* Oct 2001;11(7):587-596.

**212.** Kurnik D, Qasim H, Sominsky S, et al. Effect of the VKORC1 D36Y variant on warfarin dose requirement and pharmacogenetic dose prediction. *Thromb Haemost.* Oct 2012;108(4):781-788.

**213.** Kringen MK, Haug KB, Grimholt RM, et al. Genetic variation of VKORC1 and CYP4F2 genes related to warfarin maintenance dose in patients with myocardial infarction. *J Biomed Biotechnol.* 2011;2011:739751.

**214.** Innocenti F, Undevia SD, Iyer L, et al. Genetic variants in the UDP-glucuronosyltransferase 1A1 gene predict the risk of severe neutropenia of irinotecan. *Journal of clinical oncology : official journal of the American Society of Clinical Oncology.* Apr 15 2004;22(8):1382-1388.

**215.** Takano M, Kato M, Yoshikawa T, et al. Clinical significance of UDP-glucuronosyltransferase 1A1*6 for toxicities of combination chemotherapy with irinotecan and cisplatin in gynecologic cancers: a prospective multi-institutional study. *Oncology.* 2009;76(5):315-321.

**216.** Culley CL, Kiang TK, Gilchrist SE, Ensom MH. Effect of the UGT1A1*28 allele on unconjugated hyperbilirubinemia in HIV-positive patients receiving Atazanavir: a systematic review. *The Annals of pharmacotherapy.* Apr 2013;47(4):561-572.

**217.** Ferraris L, Vigano O, Peri A, et al. Switching to unboosted atazanavir reduces bilirubin and triglycerides without compromising treatment efficacy in UGT1A1*28 polymorphism carriers. *J Antimicrob Chemother.* Sep 2012;67(9):2236-2242.

**218.** Park WB, Choe PG, Song KH, et al. Genetic factors influencing severe atazanavir-associated hyperbilirubinemia in a population with low UDP-glucuronosyltransferase 1A1*28 allele frequency. *Clin Infect Dis.* Jul 1 2010;51(1):101-106.

**219.** Lankisch TO, Moebius U, Wehmeier M, et al. Gilbert's disease and atazanavir: from phenotype to UDP-glucuronosyltransferase haplotype. *Hepatology.* Nov 2006;44(5):1324-1332.

**220.** Panagopoulos P, Paraskevis D, Katsarolis I, et al. High prevalence of the UGT1A1*28 variant in HIV-infected individuals in Greece. *Int J STD AIDS.* Oct 2014;25(12):860-865.

**221.** Ribaudo HJ, Daar ES, Tierney C, et al. Impact of UGT1A1 Gilbert variant on discontinuation of ritonavir-boosted atazanavir in AIDS Clinical Trials Group Study A5202. *J Infect Dis.* Feb 1 2013;207(3):420-425.

**222.** Lubomirov R, Colombo S, di Iulio J, et al. Association of pharmacogenetic markers with premature discontinuation of first-line anti-HIV therapy: an observational cohort study. *J Infect Dis.* Jan 15 2011;203(2):246-257.

**223.** Rotger M, Taffe P, Bleiber G, et al. Gilbert syndrome and the development of antiretroviral therapy-associated hyperbilirubinemia. *J Infect Dis.* Oct 15 2005;192(8):1381-1386.

**224.** Metalidis C, Lerut E, Naesens M, Kuypers DR. Expression of CYP3A5 and P-glycoprotein in renal allografts with histological signs of calcineurin inhibitor nephrotoxicity. *Transplantation.* May 27 2011;91(10):1098-1102.

**225.** Hesselink DA, van Schaik RH, van der Heiden IP, et al. Genetic polymorphisms of the CYP3A4, CYP3A5, and MDR-1 genes and pharmacokinetics of the calcineurin inhibitors cyclosporine and tacrolimus. *Clinical pharmacology and therapeutics.* Sep 2003;74(3):245-254.

**226.** Uesugi M, Masuda S, Katsura T, Oike F, Takada Y, Inui K. Effect of intestinal CYP3A5 on postoperative tacrolimus trough levels in living-donor liver transplant recipients. *Pharmacogenetics and genomics.* Feb 2006;16(2):119-127.

**227.** Zhao Y, Song M, Guan D, et al. Genetic polymorphisms of CYP3A5 genes and concentration of the cyclosporine and tacrolimus. *Transplant Proc.* Jan-Feb 2005;37(1):178-181.

**228.** Haas DM, Quinney SK, Clay JM, et al. Nifedipine pharmacokinetics are influenced by CYP3A5 genotype when used as a preterm labor tocolytic. *Am J Perinatol.* Apr 2013;30(4):275-281.

**229.** Benkali K, Rostaing L, Premaud A, et al. Population pharmacokinetics and Bayesian estimation of tacrolimus exposure in renal transplant recipients on a new once-daily formulation. *Clin Pharmacokinet.* Oct 2010;49(10):683-692.

**230.** Min SI, Kim SY, Ahn SH, et al. CYP3A5 *1 allele: impacts on early acute rejection and graft function in tacrolimus-based renal transplant recipients. *Transplantation.* Dec 27 2010;90(12):1394-1400.

**231.** Niioka T, Satoh S, Kagaya H, et al. Comparison of pharmacokinetics and pharmacogenetics of once- and twice-daily tacrolimus in the early stage after renal transplantation. *Transplantation.* Nov 27 2012;94(10):1013-1019.
